# Advanced Flooring Technologies for Industrial and Commercial Buildings: A Critical Review of Materials, Performance, Durability, Design and Sustainability

**DOI:** 10.3390/polym18212717

**Published:** 2026-11-02

**Authors:** Artemis Kontiza, Maria Pastrafidou, Ioannis Kartsonakis

**Affiliations:** 1 Sika Hellas ABEE, Protomagias 15, 14568 Kryoneri, Greece; kontiza.artemis@gr.sika.com or kontizaap@mail.ntua.gr; 2 School of Applied Mathematical and Physical Sciences, Department of Mechanics, National Technical University of Athens, 15780 Athens, Greece; 3 Laboratory of Physical Chemistry, School of Chemistry, Aristotle University of Thessaloniki, 54124 Thessaloniki, Greece; mpastraa@chem.auth.gr

**Keywords:** industrial flooring, commercial flooring, epoxy flooring, polyurethane flooring, PMMA, polyaspartic coatings, cementitious flooring, hybrid flooring systems, durability, failure mechanisms, life cycle assessment, sustainability

## Abstract

Industrial and commercial flooring systems are multifunctional engineered systems exposed to mechanical, thermal, chemical, and environmental actions. This critical review compares cementitious, epoxy, polyurethane (PU), polymethyl methacrylate (PMMA), polyaspartic polyurea, and hybrid PU–cement technologies with respect to material configuration, curing, mechanical performance, durability, installation constraints, and sustainability. Evidence is interpreted according to formulation, thickness, substrate, test method, curing, and exposure conditions. Abrasion, interfacial debonding, moisture-related deterioration, thermal and ultraviolet ageing, chemical attack, and application-specific selection are examined. Emerging smart, self-healing, and digital approaches are presented as research directions whose flooring-specific validation remains limited. No technology is universally superior; selection must be based on service-specific loading, exposure, substrate condition, downtime, maintenance, and life-cycle requirements.

## 1. Introduction

### 1.1. Importance of Flooring Systems

Industrial and commercial flooring systems constitute one of the most critical components of modern buildings, serving not only as load-bearing surfaces but also as multifunctional engineering systems that directly influence structural performance, operational safety, maintenance requirements, hygiene, and overall life-cycle costs. Unlike conventional residential floors, industrial flooring is continuously exposed to severe service conditions, including heavy static and dynamic loads, abrasion, material-handling vehicle traffic, impact, chemical spills, thermal gradients, moisture ingress, and aggressive cleaning procedures. The cumulative effect of these stresses can progressively induce surface wear, cracking, erosion, loss of adhesion, and other forms of deterioration, ultimately reducing the functional performance and service life of the flooring system [1]. Consequently, industrial flooring systems require materials and surface treatments that provide high mechanical durability, abrasion and impact resistance, low permeability, and adequate resistance to chemical attack, while maintaining their mechanical integrity, dimensional stability, and functional performance over extended service periods with minimal maintenance interventions and operational interruption [2,3].

Determining the appropriate flooring specification has therefore evolved into a multidisciplinary engineering process involving polymer chemistry, structural design, materials science, durability engineering, concrete technology, occupational safety, and environmental sustainability. Depending on the intended application, flooring systems may also be required to provide additional functionalities such as electrostatic discharge (ESD) protection, anti-slip performance, waterproofing, chemical containment, antimicrobial activity, thermal insulation, decorative appearance, or rapid return to service [4]. These increasingly demanding performance requirements have driven the development of advanced flooring technologies that combine high-performance binders, fiber reinforcement, engineered aggregates, functional additives, and optimized installation techniques. Accordingly, modern flooring design is increasingly based on application-specific material selection, in which the composition and architecture of the flooring system are tailored to the anticipated mechanical loads, thermal conditions, chemical exposure, hygiene requirements, and specialized functional demands of the service environment [5,6,7].

A flooring system’s financial impact goes far beyond its upfront installation price. In industrial facilities, flooring failures may interrupt production processes, compromise worker safety, increase maintenance expenditures, damage sensitive equipment, and reduce operational efficiency. For sectors such as pharmaceutical manufacturing, food processing, logistics, healthcare, electronics, and clean-room environments, flooring performance is directly associated with regulatory compliance, product quality, and hygiene standards [8]. Consequently, selecting a flooring system based solely on initial cost rather than long-term performance often results in increased life-cycle expenses and premature rehabilitation. Therefore, life-cycle cost assessment, operations, incorporating installation, maintenance, replacement, repair, and end-of-life costs, provides a more comprehensive basis for comparing flooring alternatives and identifying the most economically sustainable solution over the intended service life [9,10].

The range of flooring technologies has expanded through advances in cementitious and polymer materials. Resistance to wear, chemicals, UV radiation, moisture, and thermal cycling is not a collective property of all polymer floors; it depends on resin chemistry, additives, filler content, layer thickness, curing, substrate condition, and service exposure. Accordingly, performance statements in this review are treated as formulation- and condition-specific [9,10,11,12].

Taking into account that modern infrastructure continues to evolve toward digitalization and sustainable asset management, flooring systems are also expected to integrate advanced functionalities, including embedded sensing technologies, predictive maintenance, structural health monitoring, and intelligent surface management [11]. These developments highlight the transition of flooring systems from passive construction materials to active engineering components capable of supporting the operational resilience and sustainability objectives of contemporary industrial and commercial buildings. In particular, the integration of self-powered sensors, real-time data acquisition, and artificial intelligence can enable flooring surfaces to continuously collect and interpret operational information, providing a technological basis for smart-building monitoring, automated decision-making, and integration with digital-twin platforms [12,13].

### 1.2. Evolution of Flooring Technologies

The development of flooring technologies has closely aligned with the evolution of construction materials and industrial requirements. Early flooring systems were primarily based on natural materials such as timber, stone, and clay, selected mainly for their local availability and adequate load-bearing capacity. Despite the fact that these materials provided acceptable structural performance for residential and public buildings, they exhibited significant limitations in terms of durability, hygiene, maintenance, and resistance to aggressive service environments [14]. The rapid industrialization of the nineteenth and twentieth centuries created the need for flooring systems capable of supporting substantially higher mechanical loads while maintaining long-term performance under continuous industrial operation. This increasing demand for strength, crack control, durability, and load-bearing capacity progressively established concrete, and subsequently reinforced and fiber-reinforced concrete, as a principal material platform for modern industrial flooring systems [15,16].

The introduction of Portland cement fundamentally transformed flooring construction by enabling the widespread use of concrete slabs characterized by structural continuity, high compressive strength, and relatively low construction costs. Concrete rapidly became the dominant flooring material for warehouses, transportation facilities, manufacturing plants, and commercial buildings. However, despite its excellent compressive behavior, conventional concrete remained susceptible to surface dusting, shrinkage cracking, abrasion, moisture penetration, and chemical degradation. These limitations stimulated extensive research into cementitious toppings, surface hardeners, polymer-modified mortars, and protective coatings designed to enhance the durability and functionality of concrete floors [17]. In particular, surface modification and protective treatments can substantially improve abrasion and chemical resistance while reducing permeability, thereby extending the service life of concrete flooring exposed to demanding industrial environments [18,19].

During the second half of the twentieth century, advances in polymer chemistry introduced a new generation of resin-based flooring systems capable of addressing many of the deficiencies associated with conventional concrete. Epoxy resins were among the first polymeric materials widely adopted for industrial flooring because of their excellent adhesion to concrete substrates, high compressive strength, superior chemical resistance, and seamless surface finish. Their ability to withstand heavy mechanical loading while maintaining hygienic, dust-free, and chemically resistant surfaces made epoxy flooring particularly attractive for pharmaceutical facilities, hospitals, laboratories, food processing plants, and manufacturing industries [20,21].

Advances in PU formulations have substantially expanded the performance capabilities of resin-based flooring, enabling systems with enhanced flexibility, impact resistance, crack-bridging capacity, and tolerance to thermal and mechanical stresses. Unlike relatively rigid epoxy systems, PU flooring can better accommodate cyclic loading and substrate movements, making it particularly suitable for facilities exposed to thermal fluctuations, vibration, and dynamic mechanical stresses. The combination of polymeric flexibility with high thermal and mechanical resistance is particularly advantageous in industrial environments where flooring is subjected simultaneously to heavy traffic, localized mechanical loads, and rapid temperature variations [22]. PU–cement hybrid systems further extend this performance by integrating the mechanical robustness and dimensional stability of cementitious components with the resilience and chemical resistance of polymer matrices, resulting in flooring systems suitable for demanding industrial service conditions [23,24].

The increasing emphasis on rapid project completion and reduced operational disruption has driven the adoption of fast-curing flooring technologies, particularly polymethyl methacrylate (PMMA) and polyaspartic systems. These materials are characterized by rapid curing kinetics, enabling commercial and industrial facilities to return to service within only a few hours after application. PMMA-based flooring systems, in particular, can achieve sufficiently rapid hardening to permit renewed service within approximately 2–3 h, substantially reducing downtime compared with conventional thermosetting flooring technologies [25]. Their combination of rapid polymerization, durability, high abrasion resistance, and applicability over a broad temperature range makes these technologies particularly attractive for logistics centers, cold-storage facilities, transportation infrastructure, retail environments, and refurbishment projects where installation windows are highly constrained.

The evolution of flooring technology is increasingly shaped by the convergence of sustainability principles, advanced materials engineering, and emerging digital technologies. Modern flooring systems increasingly incorporate recycled aggregates, supplementary cementitious materials, bio-based polymers, low-VOC (volatile organic compound) formulations, and renewable raw materials to reduce their environmental impact while maintaining high levels of mechanical performance. Simultaneously, advances in nanotechnology have enabled the incorporation of nanoparticles such as graphene derivatives, nanosilica, titanium dioxide, carbon nanotubes (CNTs), and nanoclays to improve abrasion resistance, self-cleaning properties, crack control, antimicrobial activity, and long-term durability. At relatively low addition levels, engineered nanomaterials can modify interfacial interactions and microstructural characteristics, providing a route toward multifunctional cementitious and polymer-based flooring materials with enhanced mechanical, barrier, and functional performance [26].

Digital transformation is also reshaping the flooring sector. Building information modeling, sensor-integrated flooring, Internet of Things (IoT) platforms, artificial intelligence (AI), and predictive maintenance algorithms are gradually becoming integral components of modern facility management strategies. Rather than functioning solely as passive structural surfaces, advanced flooring systems are increasingly expected to contribute to structural health monitoring, energy efficiency, safety management, and real-time operational decision-making [12,13,27].

Overall, the historical evolution of flooring technologies reflects a progressive transition from conventional load-bearing surfaces toward multifunctional engineered systems that integrate structural performance, durability, sustainability, and digital intelligence. This transition has significantly broadened the design possibilities available to engineers while simultaneously increasing the complexity of material selection and performance assessment. Consequently, understanding the advantages, limitations, and application domains of each flooring technology has become essential for optimizing both technical performance and long-term economic sustainability [10,15].

### 1.3. Challenges in Industrial and Commercial Flooring Applications

The design and selection of flooring systems for commercial and industrial buildings represent a complex engineering challenge due to the wide range of operational conditions encountered across different facilities. Unlike residential flooring, industrial floors are continuously subjected to combinations of static and dynamic mechanical loads, abrasive wear, thermal variations, chemical exposure, moisture ingress, impact loading, and repetitive traffic generated by forklifts, automated guided vehicles, heavy machinery, and pedestrian movement. The simultaneous interaction of these loading mechanisms often accelerates material degradation and significantly influences the long-term serviceability of flooring systems [7,28].

Mechanical deterioration remains a major challenge in industrial flooring due to repetitive traffic, impact, localized loading, and abrasive wear. These actions may progressively induce surface damage, cracking and loss of serviceability, as discussed in detail in Section 4 [29]. Chemical durability constitutes another critical performance requirement, particularly in manufacturing industries, food processing plants, pharmaceutical facilities, laboratories, and chemical storage areas. Exposure to acids, alkalis, oils, solvents, disinfectants, cleaning agents, and industrial chemicals may progressively deteriorate both cementitious and polymeric flooring systems through mechanisms such as chemical attack, hydrolysis, oxidation, plasticization, or loss of interfacial adhesion. Consequently, flooring materials must be carefully selected according to the specific chemical environment expected during operation [30,31].

Environmental conditions further complicate flooring performance. Moisture migration from concrete substrates, UV radiation, freeze–thaw cycles, thermal shock, elevated temperatures, and repeated temperature fluctuations may induce dimensional instability, loss of adhesion, cracking, discoloration, or premature ageing. These degradation mechanisms become particularly critical in outdoor facilities, food processing industries, refrigerated warehouses, and industrial environments characterized by significant thermal gradients. In addition to mechanical and environmental performance, modern flooring systems must increasingly satisfy sustainability objectives. Reducing embodied carbon, extending service life, minimizing VOC emissions, facilitating maintenance, and enabling material recycling have become central considerations during flooring design. Life cycle assessment (LCA), Environmental product declarations, and circular economy principles are therefore gaining increasing importance as complementary decision-making tools alongside traditional engineering criteria [23,32].

Finally, the rapid adoption of digital construction technologies introduces new expectations regarding flooring functionality. Emerging concepts such as smart floors, wireless monitoring, embedded sensing systems, digital twins, and AI-assisted predictive maintenance are transforming flooring systems into active components of intelligent infrastructure capable of continuously monitoring their own condition and supporting data-driven facility management. The combination of these technical, environmental, economic, and sustainability challenges demonstrates that flooring system selection can no longer rely solely on conventional engineering practice or initial construction cost. Instead, it requires a holistic understanding of material behavior, long-term durability, installation technology, environmental performance, and future operational requirements, which constitutes the primary motivation for the present review [2,33].

### 1.4. Scope and Objectives of the Review

This review integrates literature on load-bearing cementitious floors, screeds and toppings, protective resin coatings and mortars, resilient coverings, and hybrid systems. Its specific contribution is to compare material chemistry, engineering function, layer configuration, deterioration mechanisms, application requirements, and sustainability within one performance-based framework. The review also distinguishes flooring-specific evidence from broader coating or polymer-composite studies and identifies limitations caused by non-comparable test methods, configurations, and exposure conditions.

### 1.5. Review Methodology

A structured critical narrative-review approach was used. The bibliography assembled for the manuscript was screened for relevance to industrial and commercial flooring, concrete floor construction, resin coatings and toppings, adhesion, abrasion, chemical and thermal exposure, failure mechanisms, life-cycle assessment, and emerging smart-material concepts. Peer-reviewed studies, standards, authoritative technical guidance, and reference books were considered. Priority was given to flooring-specific studies and standardized test evidence. Broader coating and polymer-composite studies were retained only when they supported a clearly identified mechanism or emerging concept and were described as indirect evidence. Evidence was compared according to material configuration, thickness, substrate, curing and exposure conditions, test method, and laboratory or field relevance. A reproducible database-by-database search log and formal risk-of-bias assessment were not used; therefore, this article is presented as a critical narrative review rather than a systematic review.

## 2. Classification of Flooring Systems

### 2.1. General Classification

For engineering purposes, flooring systems are classified first by function and layer configuration: (i) load-bearing structural slabs; (ii) non-structural screeds and toppings; (iii) bonded protective coatings, self-leveling layers, broadcast systems, and resin mortars; and (iv) resilient, modular, or decorative coverings. Each group can then be subdivided by binder chemistry and intended application. This hierarchy avoids direct equivalence between structural concrete, thin protective coatings, and resilient finishes. Selection remains application-specific and must consider loading, substrate, chemical and thermal exposure, moisture, installation time, maintenance, and expected service life [2,22,25].

Based on their structural function, flooring systems can be broadly classified as either load-bearing systems or protective surface systems. Load-bearing systems, such as reinforced concrete slabs and cementitious screeds, primarily contribute to the structural capacity of the building while simultaneously providing the substrate for subsequent finishing layers. Conversely, resin-based coatings, self-leveling compounds, and resilient floor coverings primarily function as protective and functional surface layers that enhance durability, hygiene, safety, and operational performance without significantly contributing to the structural load-carrying capacity. Recent developments in materials engineering have further blurred the distinction between these categories through the emergence of multifunctional hybrid systems combining inorganic and organic constituents. Polymer-modified cementitious materials, fiber-reinforced overlays, PU–cement systems, and nanomodified composites represent examples of advanced flooring technologies specifically engineered to exploit the complementary advantages of different material classes. These hybrid systems typically exhibit improved adhesion, superior chemical durability, enhanced crack resistance, and extended service life compared with conventional single-component flooring systems [3,18,34].

Environmental sustainability has emerged as an increasingly important criterion for the classification and selection of modern flooring systems. Flooring materials are increasingly evaluated according to embodied carbon, VOC emissions, recycled content, recyclability, maintenance requirements, and overall life-cycle environmental impact. Life-cycle assessment (LCA) studies demonstrate that meaningful comparisons between flooring alternatives should consider not only the production stage but also installation, maintenance, replacement, service life, and end-of-life scenarios. Consequently, modern flooring classification extends beyond conventional engineering performance to incorporate circular-economy principles and life-cycle-based environmental assessment methodologies.

**Figure 1:**
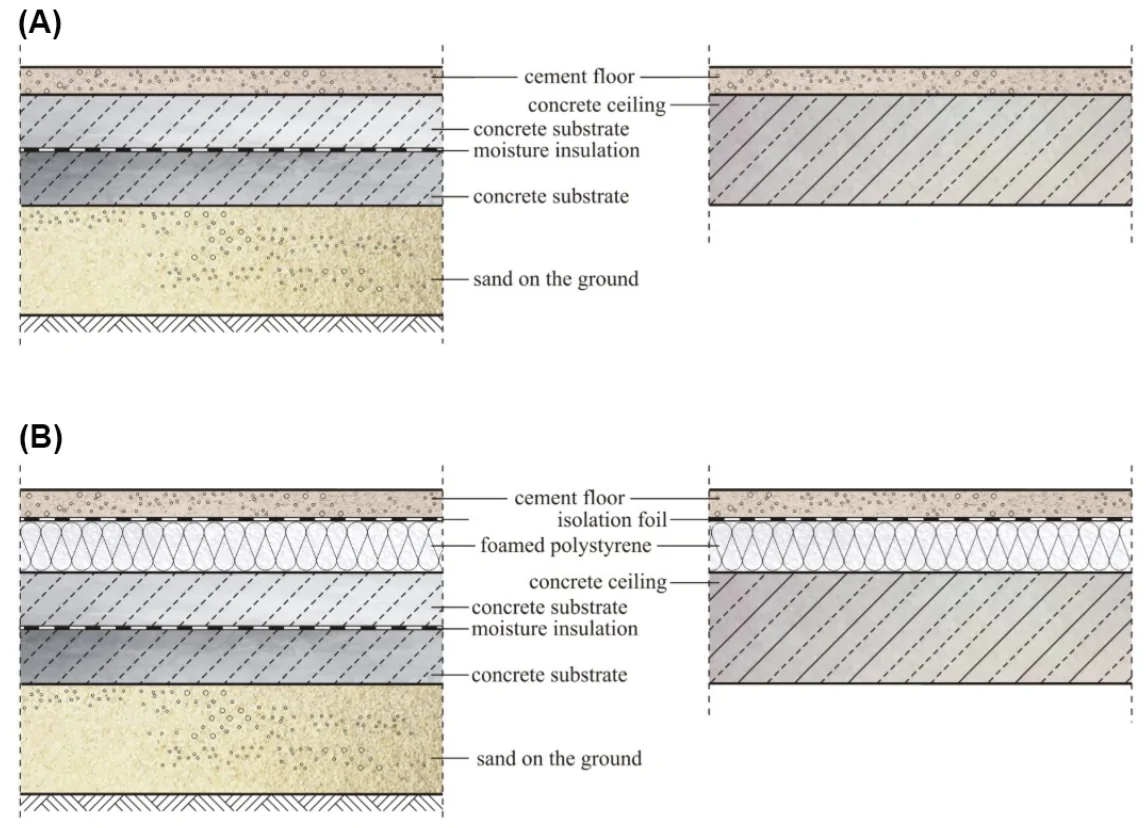
Schematic representation of different configurations of floor systems constructed on (**A**) a concrete substrate/reinforced-concrete ceiling and (**B**) an insulation layer: reproduced from [35], MDPI, 2020.

Cement-based flooring systems can perform different functions depending on their configuration within the overall floor assembly. They may constitute the final exposed surface or serve as a load-bearing substrate for subsequent functional layers, such as epoxy resin coatings. This layered approach enables the structural capacity of cementitious materials to be combined with the enhanced durability, chemical resistance, and surface functionality provided by polymer-based finishes (Figure 1) [35]. The principal categories of flooring systems discussed throughout this review, as well as their main characteristics, advantages, limitations, and typical application fields, are comparatively presented in Table 1.

### 2.2. Cementitious Flooring Systems

Cementitious flooring systems remain the most widely used flooring solution for industrial and commercial buildings due to their high load-bearing capacity, cost-effectiveness, and compatibility with a broad range of construction methods. They form the structural foundation of most floor assemblies and, in many applications, also serve as the final wearing surface after appropriate finishing treatments. Their widespread adoption is attributed to the favorable balance between mechanical performance, material availability, ease of construction, and long-term service life. Nevertheless, the increasing performance requirements of modern industrial facilities have exposed several limitations of conventional cement-based floors, driving the development of advanced cementitious technologies with enhanced durability, abrasion resistance, and sustainability [7,10,36].

From a materials science perspective, cementitious flooring systems are composite materials consisting of hydraulic binders, aggregates, water, and a variety of mineral or chemical admixtures. Upon hydration, Portland cement undergoes a series of physicochemical reactions that produce calcium silicate hydrate gel, calcium hydroxide, ettringite, and monosulfate phases. Among these hydration products, calcium silicate hydrate constitutes the primary binding phase responsible for the mechanical strength and stiffness of hardened concrete, whereas calcium hydroxide and other crystalline phases significantly influence durability and chemical resistance. The resulting microstructure, characterized by the distribution of hydration products and capillary porosity, governs the mechanical behavior, permeability, shrinkage, and long-term performance of cementitious flooring systems [7,37].

The performance of cementitious floors is strongly dependent on the water-to-cement (w/c) ratio, aggregate grading, curing conditions, and the incorporation of supplementary cementitious materials such as fly ash, ground granulated blast-furnace slag, metakaolin, silica fume, and limestone fillers. Lower water-to-cement ratios generally produce denser microstructures with reduced permeability and improved compressive strength, although they may increase the risk of autogenous shrinkage if appropriate curing practices are not implemented. Conversely, supplementary cementitious materials contribute to pore refinement through secondary pozzolanic reactions, improving durability while simultaneously reducing the embodied carbon associated with Portland cement production [7,36].

For industrial concrete floors, long-term performance is governed to a large extent by the quality and durability of the near-surface layer rather than solely by the bulk mechanical properties of the concrete. Surface abrasion, dust generation, localized impact damage, and wear caused by continuous forklift traffic frequently determine the functional service life of the flooring system. Consequently, several surface-enhancement technologies have been developed to improve the resistance of concrete floors against mechanical deterioration. Dry-shake floor hardeners represent an important advancement in industrial concrete flooring technology. These systems consist of dry blends containing high-strength mineral aggregates, pigments, hydraulic binders, quartz, corundum, metallic aggregates, and performance-enhancing additives that are broadcast onto freshly placed concrete before final mechanical finishing. Following application, moisture from the underlying concrete initiates hydration of the hardener layer, allowing it to integrate monolithically with the concrete substrate during power troweling. Depending on the aggregate type, dry-shake systems may be classified into quartz-based, corundum-based, metallic, or synthetic aggregate hardeners [15].

The performance of dry-shake surface hardeners is strongly influenced by the type and hardness of the aggregate incorporated into the cementitious formulation, allowing their properties to be tailored to different levels of industrial service. Quartz-based hardeners provide a cost-effective improvement in wear resistance and are widely used in medium-duty industrial environments. Corundum (aluminum oxide) hardeners exhibit substantially greater hardness and abrasion resistance, making them suitable for heavy-duty applications involving continuous forklift traffic and intensive mechanical wear. Metallic hardeners can provide exceptional resistance to impact and severe mechanical loading but may be less suitable for humid or chemically aggressive environments because of the potential susceptibility of metallic constituents to corrosion.

Comparative studies of dry-shake treatments have demonstrated that aggregate composition significantly influences surface hardness and resistance to abrasion and impact, with corundum- and metallic-containing systems generally providing greater mechanical resistance than conventional quartz-based treatments [1]. The long-term performance of dry-shake systems also depends strongly on application timing, concrete moisture availability, finishing quality, and curing conditions. Inadequate application or finishing can result in surface delamination, non-uniform hardening, dusting, or premature wear.

To further enhance mechanical performance, modern cementitious flooring systems frequently incorporate discrete fibers, including steel, glass, polypropylene, basalt, carbon, and synthetic macrofibers. Fiber reinforcement improves crack resistance by bridging developing microcracks and redistributing tensile stresses within the cementitious matrix. Although fibers generally provide only modest increases in compressive strength, they substantially improve post-cracking behavior, impact resistance, fatigue performance, and toughness, thereby enhancing the durability of flooring systems subjected to repeated dynamic loading. Hybrid fiber systems combining different fiber types and aspect ratios have attracted considerable research interest because they simultaneously control both microcrack initiation and macrocrack propagation [6].

Despite their high mechanical capacity, cementitious floors remain susceptible to cracking, abrasion, moisture ingress, and physicochemical degradation, as discussed in detail in Section 4 [38]. The environmental performance of cementitious flooring can be improved through clinker reduction, supplementary cementitious materials, recycled constituents, and service-life extension, as discussed in Section 5 [39].

Cementitious flooring remains a fundamental component of industrial and commercial infrastructure, combining structural reliability, material versatility, and cost-effectiveness across a broad range of service environments. Its versatility, load-bearing capacity, and economic advantages support its continued widespread application, while ongoing innovations in cement chemistry, admixture technology, fiber reinforcement, and low-carbon binder development are progressively addressing traditional limitations related to cracking, durability, and environmental impact. Accordingly, flooring selection should increasingly follow a performance-based approach that considers mechanical loading, chemical exposure, thermal conditions, installation constraints, maintenance requirements, and expected service life as interconnected design criteria [40]. Nevertheless, in environments requiring exceptional chemical resistance, rapid installation, or seamless hygienic surfaces, polymer-based flooring systems may provide superior performance, highlighting the importance of selecting flooring materials according to application-specific service conditions rather than relying solely on conventional engineering practice. The principal constituents of cementitious flooring systems and their roles in governing mechanical, rheological, and durability-related performance are tabulated in Table 2.

Conventional reinforced concrete floors constitute the structural foundation of most industrial and commercial facilities. Typically cast directly on grade or supported by structural framing systems, these floors are designed to resist substantial static and dynamic loads while providing adequate stiffness and long-term dimensional stability. Their performance depends primarily on concrete strength class, slab thickness, reinforcement detailing, curing practice, joint layout, and subgrade preparation. Although conventional concrete provides excellent compressive strength and structural reliability, its exposed surface frequently exhibits limitations including dust generation, surface scaling, abrasion, plastic shrinkage cracking, and reduced resistance to aggressive chemicals. For this reason, untreated concrete rarely satisfies the performance requirements of modern industrial facilities where heavy traffic and severe environmental exposure are expected [15,28].

Power-troweling modifies the near-surface zone of fresh concrete by finishing and densification; it does not, by itself, increase the bulk strength of the slab. When correctly timed and combined with appropriate mixture design and curing, it can improve surface regularity, reduce near-surface porosity, and enhance resistance to dusting and abrasion. Premature or excessive finishing can trap bleed water and increase the risk of delamination or blistering [17].

Cementitious screeds are non-structural flooring layers placed over concrete slabs to achieve the required surface level, flatness, and mechanical properties before the application of final floor finishes. Depending on project requirements, screeds may function either as wearing surfaces or as substrate preparation layers. Traditional screeds generally consist of Portland cement, fine aggregates, water, and plasticizing admixtures. Modern formulations frequently incorporate polymer modifiers, shrinkage-compensating additives, fibers, and supplementary cementitious materials to improve dimensional stability, crack resistance, and bond strength. Based on their installation method, screeds are commonly classified as bonded, unbonded, floating, or heated screeds. Bonded screeds adhere directly to the concrete substrate and are typically used where maximum load transfer is required. Floating screeds are separated from the substrate by insulation layers and are primarily employed in buildings requiring thermal or acoustic insulation [41].

Self-leveling cementitious screeds are high-flow, generally non-structural layers used to create level and smooth surfaces. Their performance is governed by binder formulation, fillers, admixtures, water content, thickness, curing, and substrate bond. Minimal finishing improves installation efficiency and surface regularity but does not imply an improvement in the bulk properties of the supporting structural slab [10,20,39].

Recent advances in cement-based materials have led to the development of ultra-high-performance cementitious (UHPC) flooring and ultra-high-performance fiber-reinforced concrete flooring systems. These materials are characterized by extremely dense microstructures, compressive strengths often exceeding 150 MPa, exceptionally low permeability, and outstanding durability. UHPC flooring typically incorporates optimized particle packing, silica fume, steel or synthetic fibers, high-range water reducers, and very low water-to-binder ratios. The resulting material exhibits remarkable abrasion resistance, impact performance, freeze–thaw durability, and chemical resistance compared with conventional concrete. Although the high material cost currently limits widespread adoption, UHPC flooring has attracted increasing interest for airports, ports, heavy industrial facilities, military infrastructure, nuclear installations, and transportation hubs where exceptional durability and minimal maintenance justify the higher initial investment [42,43].

### 2.3. Resin-Based Flooring Systems

Resin-based flooring systems have become one of the most important technological developments in modern industrial and commercial construction, offering performance characteristics that often exceed the capabilities of conventional cementitious materials. Unlike concrete flooring, whose primary function is structural load-bearing, polymeric flooring systems are primarily designed to provide protective, functional, and durable surface layers capable of withstanding aggressive mechanical, chemical, and environmental conditions while maintaining superior aesthetic appearance and hygiene [23,30].

The growing adoption of resin flooring has been driven by the increasing complexity of industrial environments, where floors are required to resist continuous forklift traffic, heavy static and dynamic loads, thermal cycling, aggressive chemicals, moisture exposure, abrasion, impact loading, and stringent sanitary regulations. Industries such as pharmaceuticals, biotechnology, electronics manufacturing, food processing, healthcare, logistics, automotive production, and aerospace impose performance requirements that frequently exceed the capabilities of untreated cementitious surfaces. Consequently, resin-based systems have evolved into highly engineered multilayer assemblies capable of satisfying these demanding service conditions [3,28].

Resin-based flooring systems function primarily as bonded multilayer overlays whose long-term performance is governed by the interaction between the polymeric coating and the underlying concrete substrate. Their performance therefore depends not only on the intrinsic properties of the polymer matrix but also on substrate quality, surface preparation, adhesion mechanisms, moisture condition, curing kinetics, and compatibility between successive layers. Adequate mechanical preparation of the concrete surface is particularly important because surface roughness, porosity, cleanliness, and removal of weak surface layers strongly influence coating adhesion and resistance to interfacial debonding [44]. Modern resin flooring typically consists of a primer, one or more intermediate body coats incorporating mineral fillers or aggregates, and a protective topcoat specifically engineered to provide the required mechanical, chemical, and aesthetic properties.

The polymer matrix constitutes the principal load-transfer medium within resin flooring systems and largely determines their mechanical response, thermal performance, chemical stability, curing behavior, and long-term durability. The incorporation of mineral fillers, quartz sand, silica flour, pigments, fibers, nanoparticles, and functional additives further modifies these characteristics by enhancing stiffness, abrasion resistance, dimensional stability, thermal conductivity, electrical conductivity, or decorative appearance. Consequently, resin flooring should be regarded as composite materials rather than simple polymer coatings [20].

The mechanical response of resin-based flooring is governed by polymer-network structure and differs substantially from the predominantly mineral-based load-transfer mechanisms of cementitious flooring systems. Whereas concrete derives its load-bearing capacity primarily from compressive strength and aggregate interlock, polymeric flooring relies on cross-linked molecular networks that can provide high tensile strength, strong adhesion, controlled flexibility, and effective energy dissipation. The degree of cross-linking, molecular architecture, glass-transition temperature (T_g_), and polymer-chain mobility directly influence mechanical stiffness, fracture toughness, creep resistance, and fatigue performance. In thermosetting polymers, increasing cross-link density generally enhances stiffness, dimensional stability, and T_g_, but excessive network rigidity may restrict molecular mobility and reduce fracture toughness, illustrating the fundamental trade-off between strength and damage tolerance [45]. As a result, different resin chemistries can exhibit markedly different mechanical responses under identical loading and environmental conditions.

The durability and serviceability of resin-based flooring systems are strongly governed by the integrity of the adhesive interface established between the concrete substrate and the polymeric overlay. Successful bonding depends on mechanical interlocking, physicochemical interactions, and, in some cases, chemical bonding between the resin and the concrete surface. Accordingly, appropriate substrate preparation and control of surface and moisture conditions are essential for achieving durable interfacial bonding, as discussed further in Section 4.2.

Environmental durability varies among resin-flooring systems. Resistance to oils, fuels, acids, alkalis, salts, solvents, cleaners, moisture, and UV exposure depends on resin chemistry, cross-link density, fillers, curing, defects, concentration, temperature, exposure duration, and combined loading. Chemical-resistance statements therefore require identification of the tested medium and conditions and should not be generalized across all resin floors [22,23].

Advanced resin flooring increasingly incorporates nanomaterials and functional fillers to improve mechanical, barrier, thermal, electrical, and surface properties. Conductive fillers can additionally impart electrostatic-dissipative functionality, enabling specialized flooring for electronics manufacturing and other environments requiring ESD protection [27,36]. Further developments in multifunctional and nanostructured flooring are discussed in Section 6. The environmental performance of resin flooring depends on both the impacts associated with polymer production and the potential benefits arising from durability, reduced maintenance, and extended service life. Current approaches involving bio-based feedstocks, low-VOC formulations, recyclable polymer networks, and circular material strategies are discussed in detail in Section 5 [46].

Overall, resin-based flooring systems have transformed the design philosophy of industrial flooring by enabling the development of seamless, chemically resistant, hygienic, and highly durable surfaces specifically tailored to demanding operational environments. Nevertheless, the performance of each resin technology is strongly influenced by its molecular structure, environmental stability, curing mechanism, and installation methodology. Therefore, understanding the characteristics of individual resin families is essential for selecting the most appropriate flooring solution for a given application. The following sections critically examine the principal resin technologies currently employed in industrial and commercial flooring, beginning with epoxy-based systems, which remain the benchmark against which many other polymer flooring materials are compared [3,15]. Performance comparison between cementitious and resin flooring materials is presented in Table 3.

### 2.4. Epoxy Flooring Systems

Epoxy flooring systems represent one of the most mature and extensively investigated resin-based flooring technologies currently employed in industrial and commercial buildings. Their widespread adoption is primarily attributed to their excellent adhesion to mineral substrates, high mechanical strength, outstanding chemical resistance, low shrinkage during curing, and ability to form seamless protective surfaces. Since their commercial introduction during the mid-twentieth century, epoxy resins have become the benchmark against which most other polymer flooring systems are evaluated [18,20].

**Figure 2:**
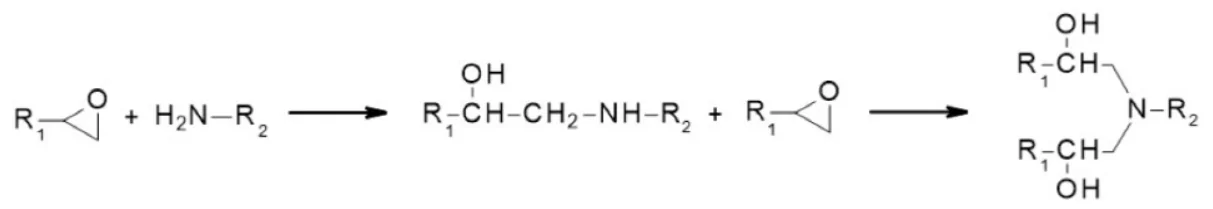
Formation and cross-linking chemistry of epoxy resins: reproduced from [48], MDPI, 2023.

Unlike thermoplastic polymers, epoxy resins belong to the family of thermosetting materials. Their final properties are developed through irreversible cross-linking reactions between epoxy functional groups and curing agents, producing a highly interconnected three-dimensional polymer network. Once cured, this network cannot be remelted or reshaped, providing exceptional dimensional stability, solvent resistance, and long-term mechanical integrity under service conditions. The density of the cross-linked network largely determines the stiffness, glass transition temperature (T_g_), chemical resistance, and fracture behavior of the resulting flooring system (Figure 2) [18,47,48].

Commercial epoxy flooring formulations generally consist of several components, including the epoxy resin, curing agent (hardener), reactive diluents, mineral fillers, pigments, rheology modifiers, defoamers, air-release agents, and performance-enhancing additives. Depending on the desired application, quartz aggregates, silica flour, colored flakes, metallic pigments, conductive particles, or decorative chips may also be incorporated to improve mechanical performance, aesthetics, or specialized functional properties [28,49].

The most employed epoxy resins for flooring applications are based on diglycidyl ether of bisphenol A, owing to their favorable balance of viscosity, mechanical properties, adhesion, and chemical resistance. Alternative resin chemistries, including bisphenol F epoxies, cycloaliphatic epoxies, novolac epoxies, and more recently bio-based epoxy resins derived from renewable feedstocks, have been developed to satisfy specific performance requirements such as enhanced chemical resistance, elevated temperature performance, reduced viscosity, or improved sustainability [47].

The curing process constitutes one of the most critical stages governing the final performance of epoxy flooring systems. Cross-linking reactions are typically initiated through the addition of amine-based curing agents, although anhydrides, polyamides, thiols, and modified cycloaliphatic hardeners are also widely employed depending on curing conditions and desired performance characteristics. The reaction kinetics are strongly influenced by temperature, resin-to-hardener ratio, humidity, and substrate conditions. Incomplete curing or improper stoichiometric proportions may significantly reduce mechanical properties, chemical resistance, and long-term durability while increasing residual stresses and susceptibility to premature failure [20,30].

At the microstructural level, cured epoxy flooring is more appropriately described as a heterogeneous particulate composite in which the polymer matrix, mineral fillers, and their interfaces collectively determine macroscopic performance. The polymer matrix continuously transfers stresses between dispersed mineral fillers and aggregates, while the filler–matrix interface plays a dominant role in determining stiffness, fracture toughness, thermal expansion, and wear resistance. Strong interfacial adhesion and an optimized particle size and distribution can promote mechanisms such as crack deflection, particle debonding, and plastic deformation of the surrounding matrix, thereby increasing the energy required for crack propagation and improving the fracture resistance of epoxy composites [50]. Proper filler dispersion minimizes local stress concentrations and restricts crack propagation, thereby improving fatigue performance and extending service life. Conversely, poor dispersion or inadequate wetting of filler particles may generate agglomerates, voids, and interfacial defects that act as preferential crack-initiation sites under mechanical loading.

Mechanical values reported for epoxy materials are configuration dependent. Unfilled cured resins, filled self-leveling toppings, aggregate-rich mortars, and complete installed systems cannot be compared as one category. Reported compressive, tensile, modulus, and adhesion values should therefore be interpreted together with resin formulation, filler fraction, specimen geometry, curing, layer thickness, substrate, and test standard [51].

A major performance advantage of epoxy flooring systems is their ability to develop strong and durable adhesion to appropriately prepared concrete substrates. Adhesion values may approach or exceed the tensile capacity of the near-surface concrete, resulting in cohesive substrate failure rather than interfacial debonding during pull-off testing. The bond strength of epoxy–concrete interfaces is strongly influenced by substrate surface characteristics, including roughness, porosity, cleanliness, and the mechanical integrity of the near-surface concrete layer. This strong interfacial bond enables effective stress transfer between the concrete substrate and the protective resin layer, reducing localized stress concentrations and contributing to long-term durability under repetitive mechanical loading.

**Figure 3:**
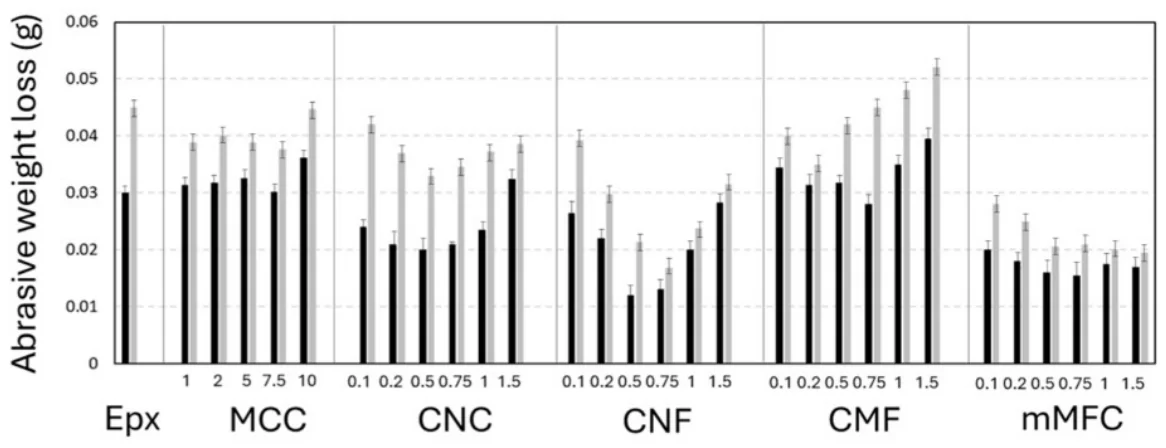
Laboratory abrasion response of cellulose-modified waterborne epoxy coatings under the loads and additive concentrations reported in the cited study. The figure provides indirect coating-level evidence and does not represent a complete industrial flooring system: reproduced from [56], MDPI, 2022.

The mechanical performance of epoxy flooring can be extensively tailored through the incorporation of mineral fillers, toughening agents, and reinforcing phases. Quartz-filled formulations generally exhibit increased stiffness, compressive strength, and abrasion resistance, whereas elastomeric modifiers can improve fracture toughness and impact resistance by increasing the material’s capacity to dissipate mechanical energy. Particulate and fibrous reinforcements can additionally activate mechanisms such as crack deflection, crack bridging, particle debonding, and matrix deformation, thereby increasing resistance to crack initiation and propagation [52]. Fiber reinforcement using glass fibers, carbon fibers, or short polymer fibers has also been investigated to improve crack resistance and fatigue behavior, although such systems remain less common in conventional flooring applications. The potential of nanoscale reinforcement to improve the wear performance of epoxy-based surfaces is illustrated in Figure 3, where appropriate nanocellulose modification substantially reduces abrasive mass loss compared with the unmodified epoxy matrix [53].

One of the defining characteristics of epoxy flooring systems is their excellent resistance to aggressive chemical environments. The highly cross-linked three-dimensional network formed during epoxy curing limits molecular mobility and reduces the penetration and diffusion of liquids and aggressive chemical species through the polymer matrix. Consequently, properly cured epoxy flooring systems provide an effective barrier against oils, fuels, dilute acids and alkalis, salts, detergents, and a wide range of industrial solvents [54]. The degree of cross-linking is therefore a critical parameter governing chemical resistance, since a denser network generally decreases the accessibility of permeating species to the underlying substrate. However, the resulting barrier performance is also strongly influenced by curing completeness, network homogeneity, porosity, and the presence of defects or microcracks that can provide preferential pathways for chemical ingress [51,55].

This chemical stability has led to the widespread adoption of epoxy flooring in pharmaceutical manufacturing, biotechnology laboratories, chemical processing plants, clean rooms, food processing facilities, and electronics manufacturing. In these environments, seamless epoxy surfaces not only resist chemical attack but also facilitate cleaning and minimize microbial contamination by eliminating joints and surface irregularities where contaminants may accumulate [30]. Nevertheless, epoxy systems are not universally resistant to all chemicals. Prolonged exposure to strong oxidizing acids, concentrated sulfuric acid, certain ketones, chlorinated solvents, and elevated temperatures may progressively degrade the polymer network through chain scission, oxidation, or plasticization, ultimately reducing mechanical integrity and adhesion [20,55].

Despite their excellent overall performance, epoxy flooring systems are subject to several degradation mechanisms that influence long-term serviceability. UV radiation represents one of the most significant environmental limitations of conventional bisphenol A epoxy systems. Continuous UV exposure induces photo-oxidation of the polymer chains, leading to discoloration, yellowing, gloss reduction, chalking, and gradual embrittlement of the surface. Although these effects generally have limited influence on structural performance, they significantly reduce aesthetic quality and may accelerate surface wear in outdoor applications. Consequently, aliphatic PU topcoats are frequently applied to improve UV resistance [30].

Thermal ageing may also alter the cross-linked network through post-curing reactions, oxidation, and thermo-mechanical stresses generated by differential thermal expansion between the resin layer and concrete substrate. Repeated thermal cycling can gradually reduce interfacial adhesion, particularly where substrate moisture or inadequate surface preparation is present [3,30].

Moisture ingress constitutes another critical degradation mechanism. Although cured epoxy exhibits relatively low water absorption compared with many other polymers, prolonged exposure to humid environments may induce plasticization of the polymer matrix, reduce stiffness and glass transition temperature, while facilitating interfacial degradation. Excessive moisture within the concrete substrate prior to installation may additionally generate osmotic blistering due to vapor pressure accumulation beneath impermeable resin layers [3,18,57].

Mechanical degradation primarily occurs through abrasion, scratching, repetitive impact, and rolling traffic. While highly filled epoxy formulations provide excellent wear resistance, localized stress concentrations generated by heavy point loads or substrate cracking may initiate brittle fracture due to the inherently limited ductility of highly cross-linked epoxy networks. Table 4 summarizes the characteristic properties of epoxy flooring systems.

Although epoxy flooring is frequently referred to as a single flooring technology, commercial epoxy flooring systems comprise a diverse family of multilayer assemblies specifically engineered to satisfy different mechanical, chemical, functional, and aesthetic requirements. The selection of an appropriate epoxy flooring configuration depends on numerous factors, including expected traffic intensity, mechanical loading, chemical exposure, substrate condition, hygiene requirements, installation time constraints, and maintenance strategies. Consequently, modern epoxy flooring should be considered a modular engineering system rather than a single material solution. In practice, epoxy flooring systems differ primarily in terms of coating thickness, aggregate content, filler concentration, curing characteristics, and overall structural architecture. Thin-film coatings are generally intended to provide surface protection and dust control, whereas thicker self-leveling systems, mortar systems, and broadcast floors are designed to withstand progressively more severe mechanical and chemical service conditions [20,28].

Thin epoxy coating systems represent the simplest and most economical epoxy flooring configuration. They are typically applied in thicknesses ranging from approximately 200 μm to 800 μm, depending on the intended service environment and number of coating layers. Their primary function is to seal the concrete surface, reduce dust formation, improve cleanability, and provide moderate resistance to chemicals and abrasion. Because of their relatively small thickness, coating systems closely follow the profile of the underlying substrate. Consequently, substrate preparation becomes particularly critical, as surface irregularities, pores, cracks, or laitance remain visible after application and may significantly influence long-term adhesion. These systems are therefore most appropriate for lightly loaded commercial facilities, storage areas, utility rooms, and maintenance spaces where structural reinforcement is not required. Despite their economic advantages, thin epoxy coatings possess limited crack-bridging capability and relatively low impact resistance. Localized substrate movement or crack formation is readily transmitted through the coating, often resulting in premature cracking or delamination [28].

Self-leveling epoxy systems constitute one of the most widely specified flooring solutions for industrial facilities requiring seamless, hygienic, and mechanically durable surfaces. These systems generally exhibit thicknesses between 2 and 5 mm and consist of low-viscosity epoxy binders combined with carefully graded mineral fillers that allow the material to flow under gravity before curing. The resulting surface exhibits excellent flatness, uniform thickness, and minimal surface roughness, facilitating cleaning while reducing bacterial accumulation and particle retention. Consequently, self-leveling epoxy flooring is extensively employed in pharmaceutical manufacturing, biotechnology laboratories, hospitals, clean rooms, electronics production facilities, and food processing plants. Mechanically, the increased thickness significantly enhances impact resistance, compressive strength, and abrasion resistance compared with thin-film coatings. Furthermore, the absence of construction joints improves hygiene while minimizing localized stress concentrations that frequently develop around conventional floor joints. However, because self-leveling systems remain relatively rigid after curing, adequate substrate stability and moisture control remain essential to prevent reflective cracking and interfacial debonding [3,57].

Among resin-based flooring technologies, epoxy mortar systems represent one of the most mechanically robust solutions for heavy-duty industrial environments subjected to severe mechanical loading and abrasion. These systems typically contain a high proportion of graded quartz aggregates, silica flour, or other mineral fillers dispersed within the epoxy matrix, producing composite materials capable of supporting extremely high compressive and impact loads. Typical installation thickness ranges from 4 to 12 mm, although greater thicknesses may be employed for specialized industrial applications. Experimental studies on epoxy mortars have demonstrated that filler content and epoxy-to-aggregate ratio strongly influence compressive and flexural strength, elastic modulus, and interfacial performance, emphasizing the importance of optimizing the particulate composition of these systems [58]. The high aggregate content substantially increases stiffness, compressive strength, abrasion resistance, and dimensional stability while reducing the volume fraction of the polymeric binder and its associated curing shrinkage.

Epoxy mortar systems are widely applied in aircraft maintenance facilities, steel processing plants, heavy manufacturing industries, loading docks, logistics centers, and areas exposed to continuous forklift traffic or concentrated mechanical loading. Their excellent resistance to localized impact also makes them suitable for environments where heavy equipment or metallic objects are frequently handled [20]. The principal disadvantage of epoxy mortar flooring lies in its relatively high installation cost and increased labor requirements associated with substrate preparation, aggregate blending, and finishing operations [18].

Quartz broadcast flooring systems combine epoxy binders with colored or natural quartz aggregates to produce highly durable surfaces exhibiting excellent slip resistance and decorative appearance. During installation, quartz aggregates are broadcast onto uncured epoxy resin until complete surface saturation is achieved. Excess aggregate is subsequently removed before application of one or more sealing topcoats [51]. The resulting composite surface provides excellent resistance to abrasion, chemical attack, and mechanical wear while simultaneously offering adjustable surface texture according to operational safety requirements. By modifying aggregate size and broadcast density, designers can optimize slip resistance for dry or wet service conditions without significantly compromising cleanability. Quartz broadcast systems are commonly specified for food manufacturing facilities, commercial kitchens, beverage production plants, parking structures, laboratories, educational buildings, and healthcare facilities where both safety and hygiene are important design considerations [18,21,30].

Decorative flake flooring systems incorporate colored polymer flakes dispersed throughout transparent or pigmented epoxy binders to produce aesthetically attractive surfaces with improved visual depth and texture. Although originally developed for commercial and architectural applications, advances in formulation have enabled decorative flake systems to achieve satisfactory mechanical performance for numerous light- and medium-duty industrial environments [20,47]. Beyond their decorative appearance, polymer flakes contribute to improved scratch concealment, enhanced slip resistance, and reduced visibility of minor surface imperfections generated during long-term service. Consequently, decorative epoxy flooring has become increasingly popular in retail facilities, educational institutions, healthcare buildings, offices, residential garages, and public buildings [28,30].

**Figure 4:**
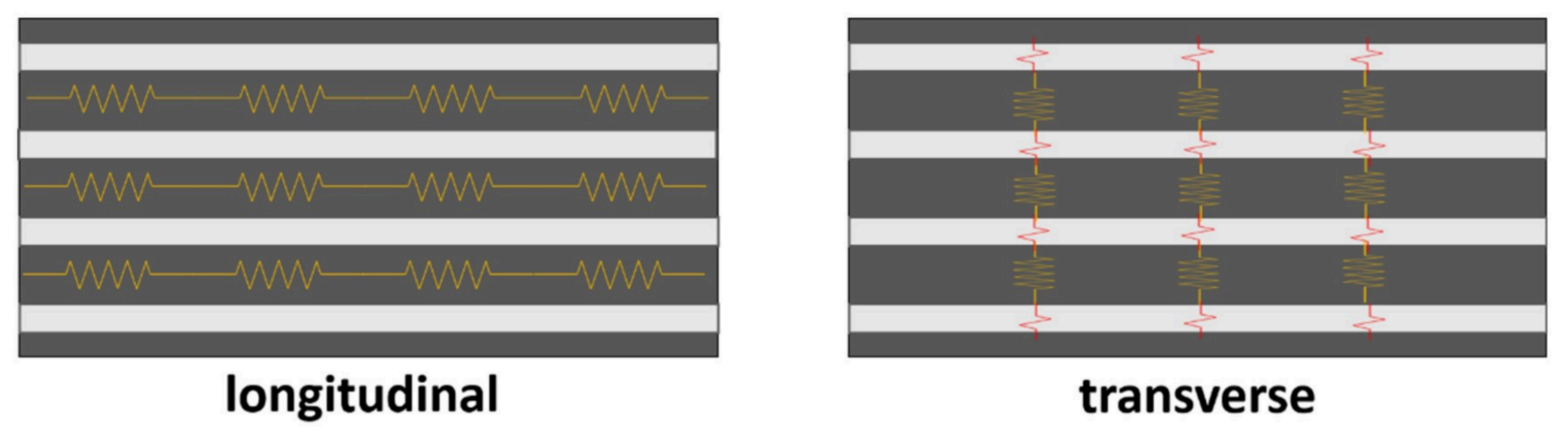
Conductive pathways in a CNT-modified polymer composite: reproduced from [59], MDPI, 2026. This schematic illustrates composite conduction and not the verified design or compliance of an installed ESD flooring system.

Electrostatic discharge (ESD) epoxy flooring represents a specialized class of functional flooring designed to safely dissipate accumulated electrical charge while retaining the mechanical and chemical performance required in industrial environments. Conventional polymer flooring generally behaves as an electrical insulator, allowing electrostatic charges generated by pedestrian movement or equipment operation to accumulate on the floor surface. Electrical conductivity can be introduced into insulating epoxy matrices through conductive fillers such as carbon black, carbon fibers, carbon nanotubes, or graphene, which form interconnected percolation networks that provide continuous pathways for charge transport [56]. In sensitive industrial environments, uncontrolled electrostatic discharge may damage electronic components, interfere with precision instrumentation, or create an ignition hazard in atmospheres containing flammable gases, vapors, or combustible dusts. The formation of interconnected conductive pathways in CNT-modified polymer composites, which provides a mechanistic basis for electrical charge transport, is schematically illustrated in Figure 4 [59].

In installed ESD flooring, conductive or dissipative behavior must be evaluated at system level, including the coating, conductive primer or network, grounding arrangement, substrate, footwear or personnel interface, and environmental conditions. CNT or carbon-filler percolation explains one conduction mechanism but does not by itself verify ESD-floor performance. Compliance should be assessed using the resistance and system-control requirements applicable to the intended facility, including ANSI/ESD S20.20 and IEC 61340-5-1 where relevant [59,60,61].

ESD epoxy flooring has become indispensable in semiconductor manufacturing, microelectronics production, aerospace industries, data centers, battery manufacturing, defense facilities, and pharmaceutical production environments where electrostatic control is essential for both equipment protection and operational safety [27,62].

The wide variety of available epoxy flooring systems demonstrates the remarkable adaptability of epoxy technology to diverse industrial requirements. Thin coatings provide economical surface protection for lightly loaded environments, whereas self-leveling systems prioritize hygiene and cleanability. Mortar systems maximize mechanical performance under extreme loading conditions, quartz broadcast floors balance durability with slip resistance, decorative flake systems emphasize architectural aesthetics, and conductive ESD floors introduce specialized electrical functionality.

Nevertheless, despite their functional differences, all epoxy flooring configurations share common limitations associated with the intrinsic characteristics of highly cross-linked thermosetting polymers, including limited strain capacity, moderate thermal shock resistance, and susceptibility to UV degradation. These limitations have stimulated the development of more flexible resin technologies, particularly PU flooring systems, which are discussed in the following section [23,63].

Although epoxy flooring systems exhibit outstanding mechanical strength, adhesion, and chemical resistance, their long-term performance is governed by complex degradation mechanisms arising from the combined effects of mechanical loading, environmental exposure, substrate characteristics, and ageing of the polymer network. In practice, flooring failures rarely originate from a single mechanism; instead, they result from the interaction of multiple physical, chemical, and mechanical processes that progressively deteriorate the integrity of the flooring system. Understanding these degradation mechanisms is therefore essential for improving service life, optimizing maintenance strategies, and developing next-generation epoxy flooring technologies [20,36]. Failure mechanisms in epoxy flooring systems may be broadly classified into interfacial failures, cohesive failures, environmentally induced degradation, and mechanically induced deterioration. The initiation and propagation of these damage modes are strongly influenced by resin formulation, cross-link density, filler morphology, curing conditions, substrate preparation, and operational loading history [49,51].

Interfacial debonding represents one of the most frequently reported causes of premature epoxy flooring failure. Because epoxy flooring functions as a bonded overlay rather than a self-supporting structural element, its long-term durability depends primarily on the quality of adhesion developed between the polymer and the concrete substrate.

Adhesion is achieved through a combination of mechanical interlocking, physicochemical interactions, and, in some cases, chemical bonding with hydrated cement phases. Surface roughness generated by shot blasting or diamond grinding allows the uncured resin to penetrate surface asperities and pores, creating mechanical anchorage after curing. Simultaneously, polar functional groups within the epoxy resin promote intermolecular interactions with hydroxyl-rich cement hydration products. However, several factors may significantly reduce bond strength. Residual laitance, dust contamination, curing compounds, oils, excessive substrate moisture, insufficient surface roughness, and improper primer application reduce effective interfacial contact and generate weak boundary layers that subsequently act as crack initiation sites [3,18].

Under mechanical loading, tensile stresses become concentrated at the resin–concrete interface. Once the local interfacial stress exceeds the adhesive strength, crack initiation occurs, followed by progressive debonding that may propagate over extensive floor areas. Depending on the relative strengths of the substrate and adhesive interface, failure may occur either as adhesive failure, where separation takes place directly at the interface, or as cohesive failure, where fracture develops within the concrete substrate or the cured resin itself [30,57]. Recent fracture mechanics studies have demonstrated that interfacial crack propagation is strongly affected by substrate roughness, moisture content, residual curing stress, and mismatch in elastic modulus between the concrete substrate and the epoxy overlay. These findings highlight that successful flooring performance depends not only on the intrinsic properties of the epoxy resin but also on the quality of substrate preparation and installation procedures [28].

Osmotic blistering is a distinctive degradation mechanism encountered in impermeable resin flooring systems applied over concrete substrates containing excessive residual moisture. Unlike conventional concrete, epoxy resins exhibit very low water permeability, preventing moisture vapor from freely escaping through the flooring layer. When soluble salts remain trapped within the concrete pores, moisture migration generates localized osmotic pressure beneath the epoxy coating. Progressive accumulation of water at the interface eventually produces localized blisters that separate the flooring from the substrate. Continued pressure development may subsequently lead to widespread delamination and complete loss of adhesion. The severity of osmotic blistering depends on substrate moisture content, permeability of the resin system, concentration of soluble contaminants, concrete age, curing conditions, and environmental temperature. Consequently, moisture testing prior to flooring installation has become an essential quality control procedure in modern resin flooring practice [64,65]. Although moisture-tolerant primers and vapor barrier systems have significantly reduced the incidence of osmotic blistering, this degradation mechanism continues to represent one of the principal causes of premature flooring rehabilitation, particularly in new concrete slabs where adequate drying periods have not been respected [3,18].

Exposure to UV radiation initiates photochemical reactions within the epoxy polymer network that progressively alter its molecular structure. UV photons possess sufficient energy to break covalent bonds within the polymer backbone, generating highly reactive free radicals that subsequently react with atmospheric oxygen. These oxidation reactions produce carbonyl and hydroxyl groups, leading to discoloration, gloss loss, chalking, and gradual embrittlement of the resin.

Although UV degradation generally affects only the outer surface of the flooring, prolonged exposure may reduce abrasion resistance and increase susceptibility to surface cracking. Consequently, conventional aromatic epoxy systems are primarily recommended for indoor applications, whereas outdoor exposure typically requires UV-stable aliphatic PU or polyaspartic topcoats. The rate of photo-oxidative degradation depends on several variables, including UV intensity, exposure duration, temperature, pigment concentration, filler content, and the incorporation of UV absorbers or hindered amine light stabilizers (HALS). Continued research into UV-resistant epoxy formulations remains an important area of polymer materials science [66].

Epoxy flooring systems are frequently exposed to repeated temperature fluctuations resulting from climatic conditions, industrial processes, refrigerated storage, steam cleaning, or thermal shock. Differences in the coefficients of thermal expansion between the epoxy overlay and the concrete substrate generate cyclic tensile and shear stresses at the interface. Repeated thermal loading accelerates fatigue damage within the polymer network and gradually weakens the adhesive bond. Simultaneously, post-curing reactions, oxidation, and physical ageing may alter the cross-link density, resulting in increased brittleness and reduced strain capacity. In facilities subjected to repeated thermal shock, these mechanisms often combine with moisture ingress and mechanical loading to accelerate crack propagation and interfacial failure [30,51].

Abrasion remains the dominant deterioration mechanism in logistics centers, manufacturing facilities, warehouses, and distribution hubs where continuous forklift traffic subjects flooring surfaces to repeated rolling and sliding contact [15,67]. Wear mechanisms include abrasive wear caused by hard particles, adhesive wear resulting from direct surface contact, fatigue wear generated by cyclic loading, and impact wear associated with falling objects. The relative importance of each mechanism depends on traffic conditions, aggregate hardness, filler morphology, surface roughness, and the mechanical properties of the cured epoxy matrix [47,66]. Quartz-filled and ceramic-filled epoxy formulations exhibit substantially improved abrasion resistance because hard mineral particles absorb a significant proportion of the applied contact stresses. Nevertheless, once wear penetrates the protective resin-rich surface layer, progressive exposure of filler particles may eventually accelerate surface deterioration [49,67].

Although epoxy flooring demonstrates excellent resistance against many industrial chemicals, prolonged exposure to highly aggressive environments may progressively alter polymer structure through hydrolysis, oxidation, plasticization, or chain scission [20,55]. Organic solvents may diffuse into the polymer network, increasing molecular mobility and reducing stiffness through plasticization. Strong oxidizing acids accelerate oxidative degradation, whereas alkaline environments may influence certain curing chemistries and filler–matrix interfaces. Elevated temperatures generally accelerate all chemical degradation processes by increasing diffusion rates and reaction kinetics. The combined action of chemical attack and mechanical loading frequently produces synergistic deterioration significantly greater than either mechanism acting independently. Consequently, future durability studies should increasingly focus on coupled degradation phenomena rather than isolated exposure conditions.

The long-term durability of epoxy flooring systems cannot be evaluated solely on the basis of intrinsic material properties. Instead, performance results from the interaction between polymer chemistry, substrate quality, environmental exposure, installation practices, and operational loading conditions. Current literature demonstrates that many premature flooring failures attributed to “material deficiencies” actually originate from inadequate surface preparation, improper curing, excessive substrate moisture, or poor construction quality [28,57].

**Figure 5:**
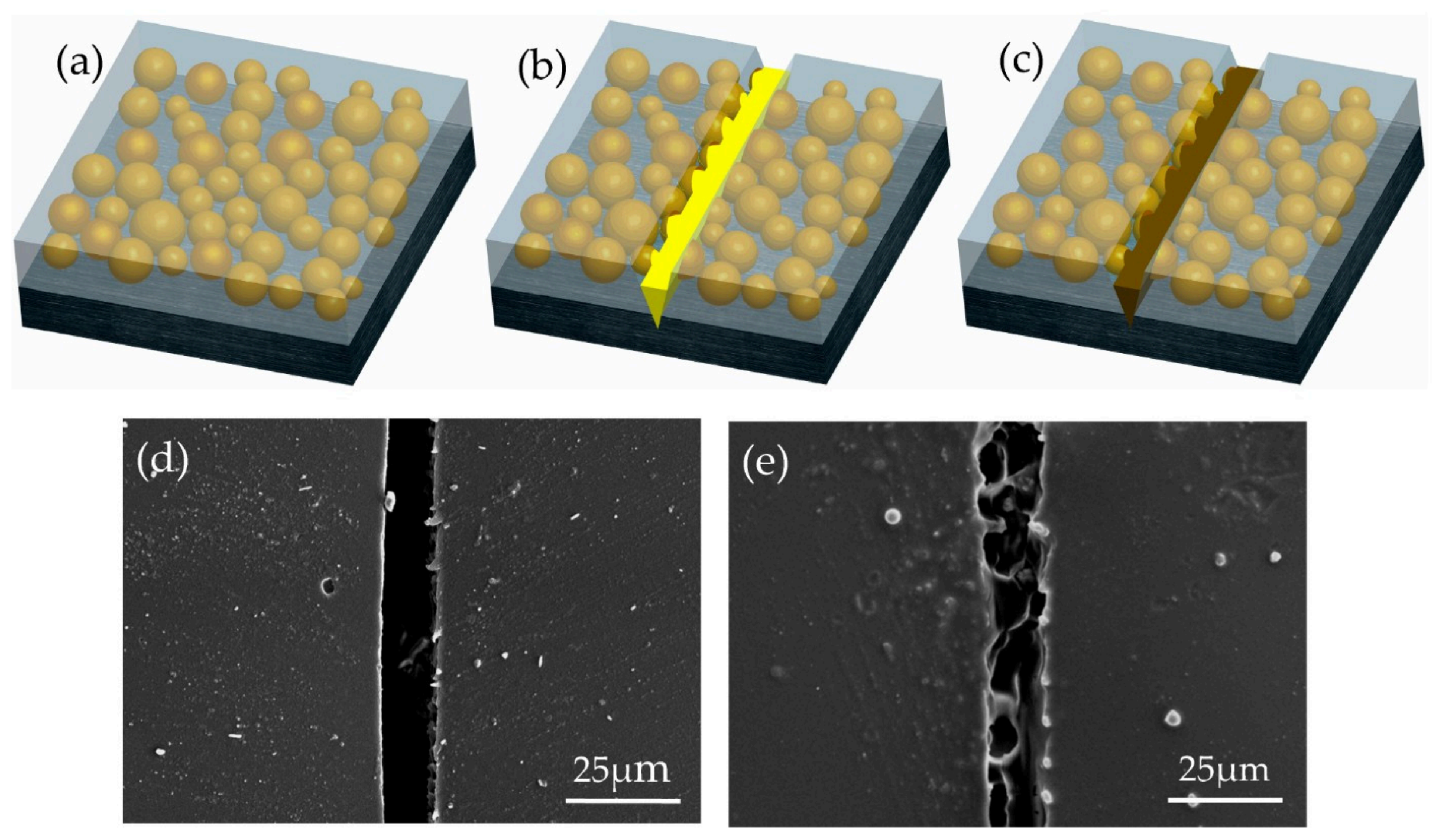
Scheme of the self-healing process. (**a**) Self-healing coating containing microcapsules on a metallic substrate. (**b**) Damage of the coating; release of the encapsulated LO on the metal surface. (**c**) A crack healed by the polymerization of LO with oxygen. SEM micrographs of the scratched area of the (**d**) pure epoxy coating and (**e**) self-healing coating with 10 wt.% microcapsules. Laboratory demonstration of a microcapsule-containing self-healing epoxy coating on a metallic substrate: reproduced from [69], MDPI, 2019. The study illustrates a healing mechanism but does not validate performance on concrete floors or under flooring service loads.

Furthermore, most published experimental studies investigate individual degradation mechanisms under controlled laboratory conditions. However, real industrial environments simultaneously expose flooring systems to cyclic mechanical loading, moisture migration, UV radiation, aggressive chemicals, and temperature fluctuations. This discrepancy highlights the need for standardized multi-factor ageing protocols capable of reproducing realistic service conditions and providing more reliable predictions of long-term flooring performance (Figure 5) [68]. The principal failure mechanisms affecting epoxy flooring systems, together with their underlying causes, microstructural effects, engineering consequences, and corresponding mitigation strategies, are summarized in Table 5.

### 2.5. Polyurethane Flooring Systems

While epoxy flooring systems continue to dominate numerous industrial applications owing to their exceptional adhesion, mechanical strength, and chemical resistance, their relatively high stiffness, limited crack-bridging capability, and sensitivity to thermal movement have stimulated the development of alternative polymer technologies. Among these, PU flooring systems have emerged as one of the most versatile and high-performance solutions for environments subjected to dynamic loading, thermal cycling, impact, and aggressive service conditions. Their unique combination of elasticity, toughness, chemical resistance, and thermal stability has resulted in widespread adoption across industries where conventional epoxy flooring may exhibit premature deterioration [63].

**Figure 6:**
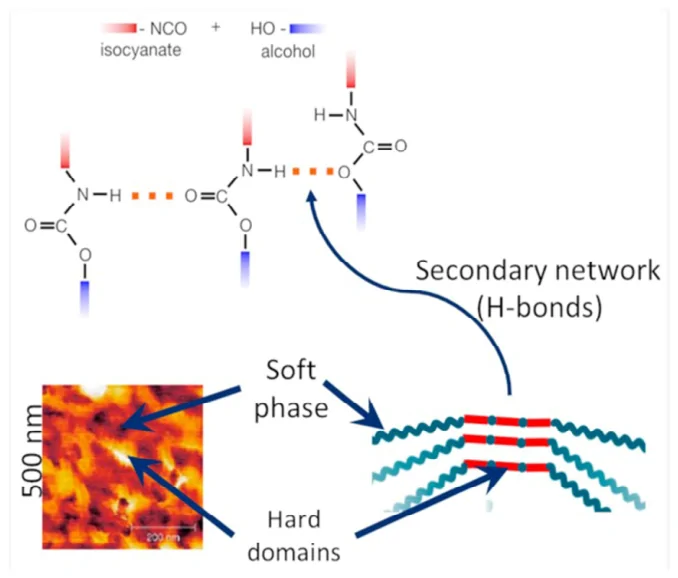
Microphase-separated structure of PU: reproduced from [71], MDPI, 2025.

Unlike epoxy resins, which form highly rigid cross-linked thermosetting networks, PU materials are synthesized through polyaddition reactions between polyols and isocyanates, producing segmented polymer structures composed of alternating soft and hard domains. The soft segments, typically derived from polyether or polyester polyols, provide flexibility, elasticity, and energy absorption, whereas the hard segments formed by the reaction of diisocyanates with chain extenders act as physical cross-links that provide strength, stiffness, and dimensional stability. The coexistence of these two phases results in a microphase-separated morphology that fundamentally governs the thermo-mechanical behaviour of PU flooring systems (Figure 6) [23,70,71].

PU behavior varies substantially with polyol and isocyanate chemistry, hard- and soft-segment composition, cross-link density, filler loading, layer configuration, and curing. Flexible coatings, elastomeric membranes, rigid PU products, and filled PU–cement composites should therefore not be treated as a single mechanical class. Microphase separation can support energy dissipation, but the resulting stiffness, strength, elongation, and durability remain formulation-specific [39].

It may be remarked that the curing process of PU flooring differs fundamentally from that of epoxy systems. Polymerization occurs through the reaction of hydroxyl groups with isocyanate groups, generating urethane linkages that progressively develop the final polymer network. The curing kinetics are influenced by temperature, humidity, catalyst concentration, and resin formulation. Moisture plays a dual role during curing: while controlled moisture can participate in specific curing mechanisms, excessive moisture may react with isocyanates to produce carbon dioxide, potentially generating bubbles, porosity, or reduced mechanical performance if installation conditions are not properly controlled. The competing reaction between isocyanate groups and water can therefore disrupt the intended polyol–isocyanate cross-linking process, emphasizing the importance of controlling substrate moisture and ambient humidity during PU coating application [72].

At the microstructural level, PU flooring can be regarded as a heterogeneous multiphase composite whose mechanical performance is governed by polymer morphology, microphase organization, filler dispersion, and interfacial interactions. Its phase-separated microstructure enhances fracture toughness by activating multiple energy-dissipation mechanisms during crack propagation, including localized plastic deformation, segmental chain mobility, crack deflection, and stress redistribution around dispersed filler particles. In particular, the hard-segment domains can act as physical cross-links that resist deformation and inhibit premature void growth, whereas the more mobile soft-segment domains accommodate large strains and dissipate mechanical energy [73]. This combination of stiffness and deformability contributes to the high resistance of PU flooring to impact loading and repeated thermomechanical stresses compared with more rigid polymer systems.

Published tensile strength, elongation, and modulus values for PU are formulation-specific and are not assigned here as a universal range for all PU floors. Flexible coatings and elastomeric products may show high deformation capacity, whereas rigid or highly filled systems and PU–cement composites exhibit different responses. Comparisons must identify formulation, filler content, specimen geometry, conditioning, temperature, and test method [74].

Thermal-shock performance of PU and PU–cement floors reflects the response of the complete layered composite, not polymer flexibility alone. Relevant factors include polymer–cement composition, aggregate and filler content, layer thickness, bond to concrete, coefficients of thermal expansion, moisture, thermal-cycle range and rate, and temperature-dependent stiffness and strength. Claims of superior performance are therefore limited to systems tested under defined cycling and bond-retention conditions [75].

Polyurethane flooring systems exhibit excellent resistance to a broad spectrum of chemicals encountered in industrial and commercial environments, including dilute acids, alkalis, oils, fats, fuels, detergents, cleaning agents, and numerous organic compounds. Their dense polymer network acts as an effective diffusion barrier, reducing the penetration of aggressive chemical species and protecting both the flooring system and the underlying concrete substrate [23,76].

The chemical stability of PU depends strongly on the chemical composition of both the polyol and isocyanate components. Polyether-based PU generally demonstrates superior hydrolytic stability and moisture resistance, making it particularly suitable for permanently wet environments such as food processing plants, breweries, dairies, and wastewater treatment facilities. In contrast, polyester-based PU often provides higher mechanical strength and abrasion resistance but is more susceptible to hydrolytic degradation when continuously exposed to moisture and elevated temperatures. The resistance of PU flooring to aggressive chemicals has resulted in widespread application in pharmaceutical manufacturing, chemical processing industries, beverage production, commercial kitchens, hospitals, and clean-room environments. Nevertheless, prolonged exposure to highly concentrated oxidizing acids, chlorinated solvents, or elevated service temperatures may gradually degrade the polymer network through oxidation, hydrolysis, or chain scission. Consequently, flooring selection should always consider the specific chemical exposure anticipated throughout the service life of the facility [23]. An important advantage of PU systems compared with conventional epoxy flooring is their superior resistance to repeated cleaning cycles involving steam, hot water, and aggressive disinfectants. This characteristic is particularly valuable in industries where hygiene regulations require frequent sanitation procedures that impose severe thermal and chemical stress on flooring materials.

The long-term performance of PU flooring is determined by the combined effects of mechanical stresses, environmental conditions, temperature fluctuations, and chemical ageing. Unlike rigid thermosetting polymers, PU possesses sufficient molecular mobility to redistribute localized stresses, thereby delaying crack initiation and reducing the likelihood of catastrophic brittle fracture. One of the primary degradation mechanisms affecting PU flooring is hydrolytic ageing, particularly in polyester-based formulations. Water molecules gradually penetrate the polymer matrix and react with susceptible ester bonds, leading to molecular chain cleavage, reduced molecular weight, and progressive deterioration of mechanical properties. Elevated temperatures significantly accelerate this degradation process by increasing diffusion rates and reaction kinetics. Hydrolytic chain scission can additionally alter the microphase-separated morphology of polyester PU, including changes in soft-segment mobility, crystallinity, and hard-domain organization, thereby progressively modifying its thermomechanical response [74].

Thermal ageing represents another important durability concern. Prolonged exposure to elevated temperatures may induce oxidation, post-curing reactions, and changes in microphase morphology, resulting in increased stiffness and reduced flexibility. Conversely, repeated freeze–thaw cycles may generate cyclic stresses at the interface between the flooring layer and concrete substrate. Owing to its comparatively low elastic modulus, PU generally accommodates these stresses more effectively than epoxy, thereby reducing the risk of interfacial debonding [18,23].

Mechanical deterioration primarily results from abrasive wear, rolling traffic, repetitive impact, and fatigue loading. Although PU exhibits excellent toughness, continuous abrasion eventually removes the polymer-rich surface layer, exposing embedded mineral fillers. The wear resistance therefore depends not only on the intrinsic properties of the polymer matrix but also on filler hardness, particle morphology, filler distribution, and interfacial bonding. UV radiation affects PU differently depending on the chemistry of the isocyanate component. Aromatic PU formulations may undergo yellowing and discoloration following prolonged UV exposure, whereas aliphatic PU exhibit significantly greater color stability and weathering resistance. For this reason, aliphatic PU topcoats are widely employed in outdoor environments where long-term color retention and aesthetic performance are essential [23]. Unlike epoxy flooring, which frequently fails through brittle crack propagation, PU flooring generally exhibits progressive damage characterized by localized plastic deformation, micro-void formation, and gradual stiffness reduction before complete failure. This damage tolerance provides an additional safety margin under severe service conditions [76].

### 2.6. Polyurethane–Cement Flooring Systems

One of the most significant developments in industrial flooring technology has been the introduction of PU–cement flooring systems. These hybrid materials combine hydraulic cement binders, PU resins, graded mineral aggregates, and functional additives into highly durable composite flooring specifically engineered for aggressive industrial environments. The performance of PU–cement flooring results from the complementary behavior of its constituent phases. The cementitious matrix contributes compressive strength, dimensional stability, and thermal compatibility with concrete substrates, while the PU component provides flexibility, impact resistance, and resistance to thermal shock. The mineral aggregates further improve abrasion resistance and reduce polymer shrinkage during curing [28].

The complementary properties of the organic and inorganic phases provide PU–cement flooring with a distinctive combination of mechanical strength, toughness, thermal stability, and chemical resistance. It is widely used as a durable flooring technology for food-processing facilities, breweries, meat-processing plants, dairy industries, commercial kitchens, pharmaceutical production, and cold-storage warehouses. A distinguishing characteristic of PU–cement systems is their ability to withstand rapid temperature changes without significant loss of adhesion. Steam cleaning, boiling water, hot-oil spills, refrigerated operation, and repeated freeze–thaw cycles generate thermal stresses that may cause premature failure in conventional flooring systems. In contrast, PU–cement flooring can accommodate these stresses more effectively owing to its balanced combination of flexibility and dimensional stability. Experimental studies have demonstrated that the mechanical response of PU–cement composites varies systematically with temperature, reflecting transitions between brittle, viscoelastic, and more ductile deformation regimes that contribute to their ability to accommodate temperature-dependent stresses [75].

Despite these advantages, PU–cement flooring requires meticulous installation procedures to ensure proper mixing, curing, and surface finishing. The complexity of installation, combined with higher material costs, remains one of the principal barriers to wider adoption in less demanding commercial applications. Nevertheless, when life-cycle performance and maintenance costs are considered, PU–cement flooring frequently represents the most economically advantageous solution for severe industrial environments [23]. Recent research has significantly expanded the capabilities of PU flooring through the incorporation of advanced functional materials and sustainable polymer chemistry. Nanostructured fillers such as nanosilica, graphene nanoplatelets, CNT, and nanoclays have demonstrated substantial improvements in wear resistance, fracture toughness, thermal conductivity, and barrier performance [77]. Their high specific surface area enables efficient stress transfer between the polymer matrix and dispersed particles, thereby delaying crack initiation and enhancing fatigue resistance.

The development of bio-based PU has also attracted considerable attention as industries seek to reduce dependence on petrochemical feedstocks. Renewable polyols derived from soybean oil, castor oil, lignin, and other biomass resources are increasingly being investigated as sustainable alternatives to conventional petroleum-derived materials. Although many bio-based formulations now achieve mechanical properties comparable to commercial products, additional research is required to fully evaluate their long-term durability under industrial service conditions. Digital technologies are expected to further transform PU flooring design and maintenance. Machine learning algorithms capable of predicting material performance, sensor-integrated flooring systems for structural health monitoring, and digital twins for maintenance optimization are emerging as promising tools for improving both service life and operational efficiency [12,13,78].

### 2.7. PMMA Flooring Systems

PMMA flooring systems can provide rapid return to service, but the terms gel time, setting, pedestrian access, readiness for mechanical loading, and full chemical service are not equivalent. Each depends on formulation, initiator and accelerator dosage, layer configuration, substrate and ambient temperature, and curing conditions. Accordingly, the 1–2 h value in Table 6 is replaced by a condition-dependent description rather than treated as a universal full-cure time [39].

PMMA flooring systems cure through free-radical polymerization of methyl methacrylate. Peroxide compounds act as radical initiators, while compatible accelerators or redox components promote radical formation at practical application temperatures; fillers, pigments, aggregates, and other additives modify application and performance but do not perform the same chemical role. Reaction rate and working time depend strongly on component dosage and temperature.

From a mechanical standpoint, PMMA flooring combines high surface hardness and abrasion resistance with sufficient toughness for demanding service conditions. Although its elastic modulus generally exceeds that of PU flooring, it remains more tolerant to thermal movement than highly cross-linked epoxy systems. Consequently, PMMA demonstrates reliable performance under cyclic loading and moderate substrate movement. One of the principal advantages of PMMA flooring is its high resistance to UV radiation and outdoor weathering. Compared with many aromatic thermosetting systems, properly formulated PMMA materials exhibit good retention of optical and surface properties during outdoor exposure. This inherent weatherability, combined with good abrasion resistance and mechanical durability, makes PMMA-based materials particularly attractive for exposed applications subjected simultaneously to solar radiation and mechanical wear [79]. This combination of properties has promoted the use of PMMA flooring in parking decks, pedestrian bridges, transportation infrastructure, balconies, and exposed industrial facilities.

PMMA flooring provides good chemical resistance to a broad range of aqueous and industrial media, including water, dilute acids and alkalis, salts, and oils. However, prolonged exposure to certain organic solvents may cause polymer swelling or softening, and careful evaluation of chemical compatibility remains essential for highly aggressive industrial environments. Experimental studies on PMMA-based composites have confirmed good resistance to water, sodium hydroxide, sodium chloride, sodium sulfate, and oils, while solvents such as acetone and toluene can produce substantially greater degradation [80]. Despite its numerous advantages, PMMA flooring presents several practical limitations. The polymerization reaction is highly exothermic and requires accurate control of catalyst dosage, ambient temperature, and working time. Furthermore, methyl methacrylate monomers generate a characteristic odour during installation, requiring adequate ventilation and appropriate occupational safety measures. Installation therefore requires experienced contractors capable of carefully controlling curing kinetics under varying environmental conditions.

Recent developments in PMMA technology have focused on reducing VOC emissions, improving fire performance, incorporating bio-derived raw materials, and enhancing mechanical properties through nanomodification using nanosilica, graphene nanoplatelets, and ceramic nanoparticles [81]. Research has also explored antimicrobial formulations and self-cleaning surface technologies for healthcare and food-processing environments. Overall, PMMA flooring represents a highly specialized flooring solution where rapid return to service, outdoor durability, and weather resistance outweigh the increased installation complexity and material cost. Its combination of rapid curing, excellent environmental durability, and high mechanical performance continues to expand its application in infrastructure rehabilitation and industrial refurbishment projects (Table 6).

### 2.8. Polyaspartic Flooring Systems

Polyaspartic flooring has emerged as an advanced class of high-performance resin systems that combines rapid curing with the durability required for demanding service environments. Originally developed as a modification of polyurea chemistry, polyaspartic coatings have rapidly gained industrial acceptance because they combine many of the advantages of epoxy and PU systems while significantly reducing curing time. Their excellent weather resistance, high abrasion performance, rapid return to service, and broad application temperature range have made them increasingly attractive for both new construction and rehabilitation projects. Polyaspartic technology is based on the reaction between aliphatic polyisocyanates and polyaspartic esters, producing highly cross-linked polyurea networks. Unlike conventional polyurea systems, whose extremely rapid reaction rates often require specialized spray equipment, polyaspartic formulations exhibit slower and more controllable curing kinetics, allowing application using conventional rollers and squeegees while maintaining excellent mechanical performance. The steric hindrance surrounding the secondary amine groups of polyaspartic esters moderates their reaction with isocyanates, enabling the curing rate and application window to be tailored from several minutes to hours [82].

From a mechanical perspective, polyaspartic flooring combines high tensile strength with excellent resistance to abrasion and impact loading. The highly cross-linked polymer network provides outstanding wear resistance while maintaining sufficient flexibility to accommodate minor substrate movements without premature cracking. Furthermore, polyaspartic coatings exhibit excellent adhesion to properly prepared concrete substrates, allowing their application as protective coatings, self-leveling systems, and decorative flooring solutions. The combination of high tensile strength and substantial elongation at break enables polyaspartic polyurea coatings to dissipate mechanical stresses while maintaining strong interfacial bonding with concrete substrates [83].

Polyaspartic polyurea coatings are based on reactions between polyaspartic esters and aliphatic polyisocyanates. Initial colour and gloss retention can be favorable compared with aromatic epoxy systems, but retained UV and abrasion performance depends on isocyanate chemistry, additives, pigments, coating thickness, curing, and exposure. Long-term photo-oxidation, gloss loss, microcracking, and surface degradation remain possible; therefore, UV stability is not treated as absolute [22].

In terms of long-term durability, polyaspartic flooring provides a robust protective barrier against moisture, abrasion, freeze–thaw exposure, chloride-containing salts, and a broad range of chemical environments. Nevertheless, long-term exposure to highly concentrated acids or aggressive organic solvents may still result in gradual polymer degradation, emphasizing the importance of proper material selection according to the intended service environment. Recent experimental studies have shown that polyaspartic polyurea coatings can retain coating integrity and adhesion after 200 freeze–thaw cycles while also providing very low chloride-ion permeability, demonstrating their suitability for concrete exposed to severe environmental conditions [82]. Despite these advantages, polyaspartic flooring systems generally remain more expensive than conventional epoxy systems. In addition, the relatively rapid curing process requires careful planning during installation to ensure uniform application and avoid visible lap marks over large floor areas. Nevertheless, the combination of rapid installation, excellent weatherability, and long-term durability continues to drive increasing adoption across numerous industrial and commercial sectors. Their main performance characteristics and typical application fields are summarized in Table 7.

### 2.9. Hybrid Flooring Systems

Hybrid flooring systems have evolved as a versatile materials-engineering approach that exploits the complementary properties of inorganic cementitious phases and organic polymer networks. Rather than relying on a single binder system, hybrid floors integrate inorganic hydraulic binders with organic polymers to produce composite materials exhibiting enhanced mechanical performance, improved durability, superior adhesion, and greater tolerance to severe industrial environments. The resulting interpenetration and interaction between the polymeric and cementitious phases can improve stress transfer, restrict crack propagation, and provide a more favorable balance between strength and deformation capacity than conventional cement-based materials [84].

Among the various hybrid flooring technologies, PU–cement and epoxy-modified cementitious systems constitute two of the most established approaches for combining polymer functionality with the structural characteristics of cement-based materials. PU–cement systems combine Portland cement, PU binders, graded mineral aggregates, and specialized fillers into dense composite structures capable of resisting simultaneous mechanical, thermal, and chemical loading. Their excellent thermal shock resistance, moisture tolerance, and chemical durability make them particularly suitable for food-processing facilities, breweries, dairies, pharmaceutical plants, and commercial kitchens where conventional resin systems may exhibit premature deterioration. Experimental investigations have confirmed that PU–cement composites can simultaneously provide high compressive, flexural, and tensile strengths together with strong adhesion to concrete and resistance to acidic and alkaline environments [85]. Epoxy-modified cementitious systems constitute another important category of hybrid flooring materials. The incorporation of epoxy polymers into cement-based matrices improves bond strength, reduces permeability, enhances abrasion resistance, and increases resistance to aggressive chemical environments while maintaining compatibility with conventional concrete substrates. These systems are frequently employed for concrete rehabilitation, industrial floor repair, and heavy-duty overlays.

Recent advances in hybrid flooring materials have increasingly focused on multiscale reinforcement and more sustainable constituent selection through the incorporation of nanomaterials, fibers, recycled aggregates, and supplementary cementitious materials. Nanosilica, graphene derivatives, carbon fibers, and basalt fibers have demonstrated the potential to improve crack resistance, wear performance, and interfacial bonding, while recycled aggregates and bio-based polymers contribute to reducing environmental impacts without significantly compromising mechanical performance. At the microstructural level, polymer modification can alter pore structure and establish polymer–cement interactions that strongly influence the mechanical behavior and transport characteristics of the resulting composite [86]. Although hybrid flooring systems generally involve higher initial installation costs and more complex application procedures than conventional cementitious floors, their extended service life and reduced maintenance requirements can potentially result in lower life-cycle costs. Consequently, hybrid technologies are increasingly recognized as a promising direction for next-generation industrial flooring.

## 3. Comparative Performance of Advanced Flooring Technologies

Flooring selection should follow a conditional decision framework rather than a universal recommendation. Minimum input criteria include structural and traffic loading, abrasion or impact demand, substrate strength and moisture, chemical type and concentration, temperature range and cycling, UV or weather exposure, slip-resistance and hygiene requirements, allowable installation downtime, maintainability, expected service life, and life-cycle cost. Candidate systems should be screened against project-specific thresholds and verified using compatible test data [9,10].

Although numerous flooring technologies have been developed to satisfy specialized industrial applications, no single material provides optimal performance under all operating conditions. Cementitious flooring systems offer excellent structural capacity and cost-effectiveness but generally require additional surface protection in chemically aggressive environments. Epoxy flooring provides outstanding adhesion, chemical resistance, and hygienic performance but remains relatively rigid and susceptible to thermal stress. Polyurethane flooring exhibits superior flexibility and thermal shock resistance, whereas PMMA and polyaspartic systems offer exceptionally rapid curing and excellent weather resistance. Hybrid flooring technologies combine the complementary advantages of inorganic and organic materials, providing balanced performance under severe industrial conditions. To facilitate engineering decision-making, the following sections critically compare the principal flooring technologies based on their mechanical behavior, durability, environmental resistance, sustainability, and overall suitability for different industrial applications [9,36].

### 3.1. Mechanical Performance

Mechanical performance remains one of the primary criteria governing flooring selection in industrial facilities. Floors are continuously subjected to compressive loads from stored materials, rolling loads generated by forklifts and automated guided vehicles, localized impact from falling objects, repetitive fatigue loading, and abrasion produced by continuous traffic. Consequently, flooring materials must exhibit not only high strength but also sufficient toughness, wear resistance, and dimensional stability throughout their service life. Conventional cementitious flooring systems provide the highest structural load-bearing capacity because of their substantial thickness and excellent compressive strength. However, their relatively brittle behaviour makes them susceptible to shrinkage cracking and localized surface deterioration under repeated impact or abrasion. Epoxy flooring systems exhibit outstanding compressive strength, surface hardness, and adhesion to concrete substrates, making them highly suitable for heavy industrial environments requiring chemically resistant seamless surfaces. Nevertheless, their relatively high stiffness limits their ability to accommodate substrate movement, increasing the likelihood of brittle cracking under thermal or mechanical deformation [30,51].

Polyurethane flooring demonstrates a markedly different mechanical response owing to its elastomeric molecular structure. Although its compressive strength is generally lower than highly filled epoxy systems, its superior flexibility, energy absorption capacity, and fatigue resistance provide significant advantages under dynamic loading conditions. This behaviour substantially reduces crack propagation and enhances long-term durability where vibration, impact, or thermal expansion are dominant design considerations. PMMA flooring provides a balanced combination of hardness, abrasion resistance, and impact performance while offering exceptionally rapid strength development after installation. Polyaspartic flooring exhibits comparable mechanical characteristics while additionally providing superior UV stability, making it particularly suitable for exposed outdoor applications. Hybrid PU–cement flooring combines the compressive strength of cementitious materials with the toughness and flexibility of PU, resulting in one of the highest overall mechanical performances among currently available industrial flooring systems [18,23].

### 3.2. Chemical Resistance

Chemical resistance is particularly important in facilities where flooring systems are routinely exposed to aggressive substances, including acids, alkalis, oils, fuels, solvents, disinfectants, and cleaning agents. Chemical degradation not only affects the surface appearance of flooring materials but may also progressively reduce mechanical integrity, adhesion, and long-term durability [51]. Cementitious flooring systems exhibit limited intrinsic resistance to aggressive chemical environments because hydration products such as calcium hydroxide readily react with acidic solutions, leading to dissolution, increased porosity, and progressive material deterioration. Consequently, unprotected concrete floors are generally unsuitable for chemical processing plants, pharmaceutical manufacturing, and food production facilities without additional protective coatings [15,28].

Epoxy flooring systems remain the benchmark for chemical resistance in industrial applications. Their highly cross-linked polymer networks effectively restrict chemical diffusion and provide excellent protection against oils, fuels, diluted acids, alkalis, detergents, and numerous industrial chemicals. However, prolonged exposure to strong oxidizing acids, chlorinated solvents, or elevated temperatures may gradually reduce polymer stability. Polyurethane flooring exhibits similarly high resistance to a wide range of industrial chemicals while providing superior performance under repeated cleaning cycles involving hot water and steam. Polyether-based formulations demonstrate particularly good hydrolytic stability, making them highly suitable for continuously wet service environments [76,87]. PMMA and polyaspartic flooring systems also provide excellent chemical durability while offering the additional benefit of rapid installation. Nevertheless, compatibility with highly aggressive chemicals should always be evaluated according to manufacturer specifications and anticipated exposure conditions. Hybrid PU–cement flooring combines the chemical stability of PU with the dimensional stability of cementitious materials, providing exceptional durability under severe chemical and thermal loading simultaneously [18,23].

The long-term performance of flooring systems is strongly influenced by environmental exposure throughout their service life. In addition to mechanical loading, industrial floors are continuously subjected to moisture, temperature fluctuations, UV radiation, freeze–thaw cycles, aggressive cleaning procedures, and chemical contaminants. The interaction between these environmental factors often governs deterioration rates and ultimately determines maintenance frequency, rehabilitation requirements, and overall service life. Consequently, environmental durability represents one of the most important criteria in the selection of flooring technologies for industrial and commercial applications [9,10].

Unlike structural mechanical properties, which can often be quantified through standardized laboratory tests, environmental durability involves complex degradation mechanisms that evolve over extended periods under combined physical and chemical exposure. Moisture diffusion, thermal expansion mismatch, polymer ageing, chemical reactions, and UV-induced degradation frequently occur simultaneously, accelerating material deterioration beyond that predicted by individual laboratory tests. Therefore, evaluating flooring performance requires consideration of the actual service environment rather than isolated material properties [36].

### 3.3. Thermal and Environmental Durability

Moisture exposure represents one of the most influential environmental factors governing the durability and service performance of flooring systems. Water ingress may weaken adhesive interfaces, promote hydrolytic degradation, increase pore saturation within cementitious materials, and accelerate freeze–thaw deterioration. Furthermore, excessive substrate moisture prior to installation is a leading cause of premature flooring failure in bonded resin systems. At epoxy–cement interfaces, the presence of water can disrupt interfacial interactions and substantially reduce adhesion, thereby increasing the susceptibility of bonded flooring systems to debonding and delamination [88]. Conventional cementitious floors exhibit relatively high permeability due to their interconnected capillary pore structure. Although supplementary cementitious materials and low water-to-cement ratios significantly reduce permeability, prolonged moisture exposure may still promote carbonation, chloride ingress, sulfate attack, and reinforcement corrosion where applicable.

Epoxy flooring demonstrates excellent resistance to liquid water because of its dense cross-linked polymer network. However, its low vapor permeability may result in osmotic blistering when applied over insufficiently dried concrete substrates. Polyurethane flooring generally exhibits greater tolerance to residual substrate moisture while maintaining excellent long-term resistance under permanently wet service conditions. Hybrid PU–cement flooring performs particularly well because it combines moisture tolerance with superior adhesion and thermal compatibility. PMMA and polyaspartic systems also exhibit excellent water resistance, although their long-term performance remains strongly dependent on substrate preparation and interfacial integrity [30,63,89].

Industrial flooring is frequently exposed to rapid temperature fluctuations generated by manufacturing processes, refrigerated storage, steam cleaning, and outdoor climatic conditions. These thermal variations produce cyclic expansion and contraction, generating tensile and shear stresses both within the flooring material and at the interface with the concrete substrate. Cementitious flooring generally exhibits excellent dimensional stability under moderate temperature variations because its coefficient of thermal expansion closely matches that of concrete. Nevertheless, restrained thermal movement may induce shrinkage cracking and joint deterioration [7,15].

Epoxy flooring provides excellent performance under stable indoor temperatures but becomes more susceptible to cracking and interfacial debonding when repeatedly subjected to severe thermal cycling due to its relatively high stiffness and limited deformation capacity [20,30,51]. Polyurethane flooring demonstrates significantly better thermal shock resistance because its elastomeric molecular structure effectively accommodates differential thermal expansion without generating excessive internal stresses. Consequently, PU and PU–cement flooring systems are widely recognized as the preferred solutions for food processing facilities, commercial kitchens, breweries, and refrigerated warehouses where repeated thermal cycling represents a dominant deterioration mechanism [23,28,63].

Outdoor flooring systems must maintain their functional and aesthetic performance under the combined effects of solar radiation, moisture, temperature variations, and atmospheric contaminants. These environmental factors gradually alter polymer chemistry, resulting in color fading, gloss reduction, oxidation, embrittlement, and surface erosion. In particular, UV irradiation can induce photolytic bond cleavage and photo-oxidation in polymeric coatings, leading to discoloration, chalking, microcracking, and progressive deterioration of surface properties [90]. Conventional cementitious flooring exhibits excellent UV stability because its mineral constituents remain largely unaffected by solar radiation. However, prolonged weathering may still contribute to carbonation, surface scaling, and freeze–thaw damage.

The resistance of resin flooring to ultraviolet exposure varies substantially with polymer chemistry and the nature of the curing network. Aromatic epoxy coatings generally exhibit relatively poor UV stability, with photo-oxidative degradation progressively promoting polymer-chain scission, yellowing, chalking, and loss of surface gloss. Consequently, epoxy flooring is generally recommended for indoor environments unless protected by UV-resistant topcoats. Polyurethane flooring demonstrates considerably improved weathering performance, particularly when based on aliphatic isocyanate chemistry. Polyaspartic flooring exhibits excellent colour stability and long-term outdoor durability, making it particularly attractive for exposed infrastructure. Accelerated UV-weathering studies of polyaspartic ester–polyurea coatings have demonstrated good color retention, while also showing that UV-A exposure can induce photo-oxidation, gloss loss, and microcracking, and that these effects can be further suppressed through incorporation of UV-protective nanoparticles [91]. PMMA systems also exhibit high weather resistance, contributing to their extensive application in parking decks, transportation infrastructure, and external walkways.

### 3.4. Hygienic and Functional Performance

A seamless and cleanable surface can support hygiene by limiting joints and facilitating cleaning, but it is not necessarily antimicrobial. Intrinsic antimicrobial activity requires a defined active mechanism and testing against relevant microorganisms under specified conditions. The two concepts are therefore treated separately.

## 4. Failure Mechanisms of Advanced Flooring Systems

Despite substantial progress in flooring materials and installation technologies, premature deterioration continues to compromise the serviceability of industrial and commercial floors. Flooring systems are exposed to a combination of mechanical loading, environmental actions, chemical attack, thermal fluctuations, and installation-related defects that progressively reduce their structural integrity and functional performance. Unlike structural failures, flooring deterioration often develops gradually through the accumulation of localized damage, eventually leading to cracking, surface wear, loss of adhesion, reduced hygiene, and costly maintenance interventions. Field investigations indicate that inadequate material selection, design detailing, construction quality, and compliance with technological requirements can initiate defects that progressively develop into cracking, delamination, and other forms of serviceability loss [92].

Failure mechanisms are rarely governed by a single deterioration process. Instead, they result from the interaction of multiple physical and chemical phenomena acting simultaneously throughout the service life of the flooring system. Mechanical abrasion may expose the polymer matrix to chemical attack, moisture ingress may weaken the flooring–substrate interface, while repeated thermal cycling accelerates fatigue crack propagation. Consequently, understanding the interaction between different degradation mechanisms is essential for predicting long-term performance and optimizing flooring design [9,36].

The principal failure mechanisms encountered in advanced flooring systems include abrasive wear, impact damage, interfacial debonding, moisture-induced deterioration, thermal cracking, UV degradation, chemical attack, fatigue damage, and joint-related failures. The severity of each mechanism depends not only on the intrinsic properties of the flooring material but also on substrate preparation, installation quality, operational loading, environmental exposure, and maintenance practices [3,30,93].

### 4.1. Mechanical Deterioration

Abrasion is one of the most common deterioration mechanisms affecting industrial flooring, particularly in warehouses, logistics centres, manufacturing facilities, airports, and distribution hubs where continuous pedestrian and vehicular traffic produces repeated surface wear [15,67,94]. Wear mechanisms are generally classified into abrasive wear, adhesive wear, erosive wear, and fatigue wear. Abrasive wear occurs when hard particles or rough surfaces remove material from the flooring surface through micro-cutting or micro-ploughing. Adhesive wear develops through repeated direct contact between opposing surfaces, whereas fatigue wear results from repeated cyclic loading that gradually initiates and propagates microcracks beneath the surface.

The abrasion resistance of flooring systems depends primarily on surface hardness, fracture toughness, filler characteristics, aggregate distribution, and interfacial bonding. Cementitious floors containing hard mineral aggregates exhibit excellent wear resistance but remain susceptible to surface dusting if improperly cured. Epoxy and polyaspartic flooring generally demonstrate superior abrasion resistance owing to their dense polymer networks, while PU flooring compensates for its slightly lower hardness through enhanced toughness and resistance to crack propagation. Recent research has demonstrated that nanostructured fillers, including nanosilica, alumina nanoparticles, graphene nanoplatelets, and silicon carbide particles, significantly improve wear resistance by increasing surface hardness and reducing microcrack formation during repeated loading [47,66,95].

### 4.2. Interfacial Failure and Delamination

Interfacial debonding represents one of the most critical failure mechanisms in bonded flooring systems because it directly compromises load transfer between the flooring layer and the concrete substrate. Unlike cohesive failure within the flooring material itself, debonding initiates at the flooring–substrate interface where stress concentrations are typically highest [3]. Adhesion is governed by three complementary mechanisms: mechanical interlocking within the substrate surface profile, physicochemical interactions between the polymer and hydrated cement phases, and chemical bonding where compatible functional groups are present. Any factor reducing the effectiveness of these bonding mechanisms may initiate premature debonding [30].

**Figure 7:**
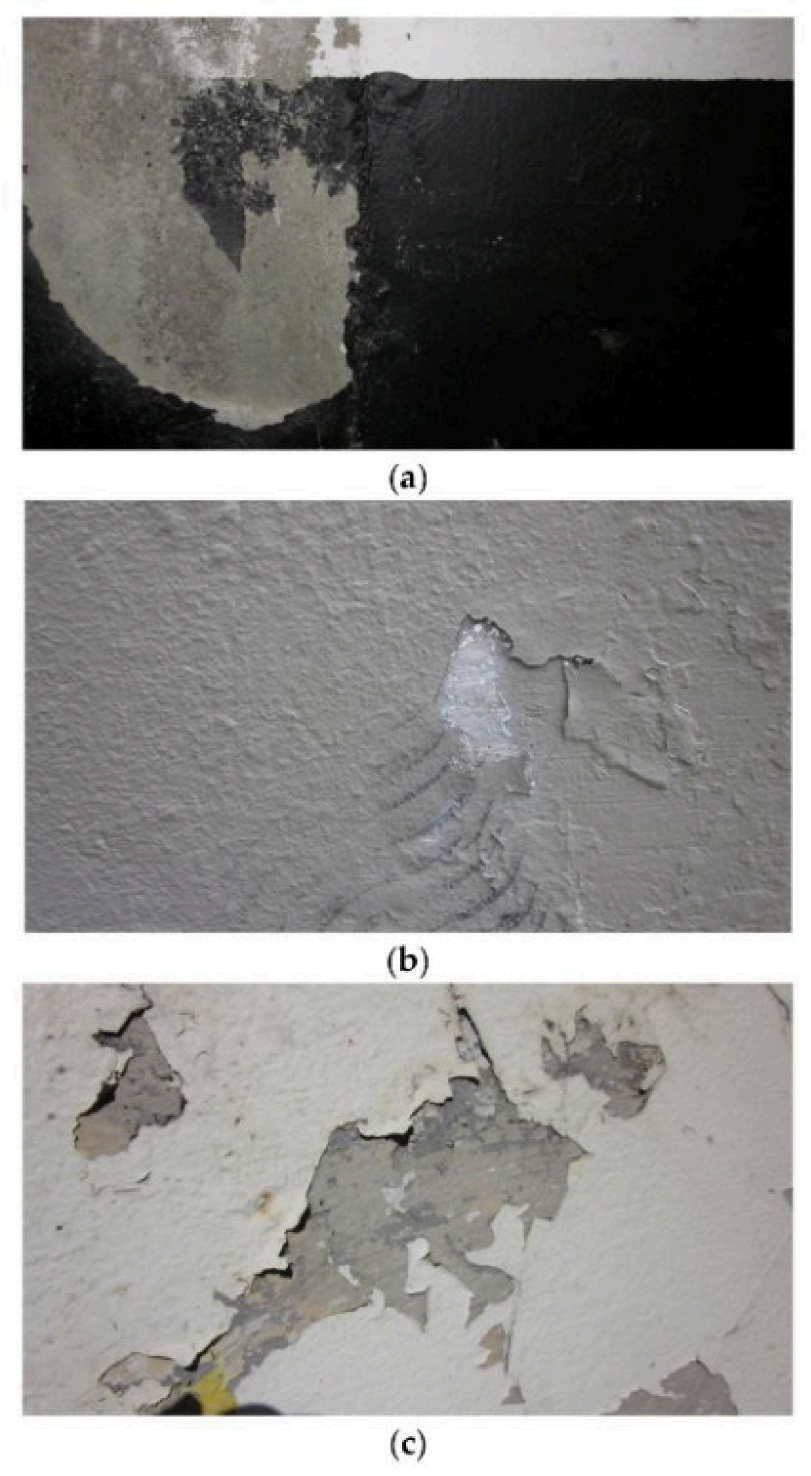
Examples of delamination observed in different protective coating systems applied to concrete: (**a**) bituminous coating, (**b**) cementitious coating, and (**c**) polymeric coating: reproduced from [96], MDPI, 2017.

Common causes include inadequate surface preparation, residual laitance, dust contamination, excessive substrate moisture, insufficient curing, incompatible primers, and improper installation procedures. Mechanical loading, thermal expansion mismatch, and moisture migration further accelerate crack propagation along the interface. Experimental investigations consistently demonstrate that proper surface preparation frequently has a greater influence on long-term flooring durability than modest differences in resin formulation, emphasizing the importance of installation quality in industrial flooring performance [18,20,57]. Typical manifestations of interfacial deterioration and delamination in different protective coating systems applied to concrete substrates are illustrated in Figure 7.

### 4.3. Moisture-Induced Degradation

Moisture-related deterioration includes distinct mechanisms that should not be used interchangeably. Moisture transport through concrete can alter interfacial conditions; vapor-pressure differences can contribute to blistering; osmotic blistering additionally requires soluble species and a concentration gradient; and water can independently reduce adhesion or promote hydrolysis in susceptible polymers. The dominant mechanism depends on substrate moisture, permeability, contaminants, temperature, coating permeability, preparation, and bond quality [32,68].

### 4.4. Thermal and UV Ageing

Industrial flooring systems often experience repeated thermal loading generated by climatic conditions, refrigerated storage, hot process equipment, steam cleaning, and direct solar radiation. These temperature variations generate expansion and contraction cycles that induce tensile stresses within the flooring layer and at the flooring-substrate interface [30,51]. Rigid flooring materials such as conventional concrete and highly cross-linked epoxy systems are generally more susceptible to thermal cracking because of their limited deformation capacity. Conversely, PU flooring accommodates differential thermal expansion through elastic deformation, thereby reducing crack initiation and improving long-term durability. Thermal fatigue becomes particularly critical in food-processing industries, breweries, commercial kitchens, and cold-storage facilities where repeated thermal shock represents a routine operational condition [23,28,63].

Chemical degradation occurs when aggressive substances interact with the flooring material, progressively altering its chemical structure and reducing its mechanical performance. The degradation mechanisms differ considerably between cementitious and polymeric flooring systems. Acidic environments primarily attack cementitious materials through dissolution of calcium hydroxide and decalcification of calcium silicate hydrate, resulting in increased porosity and strength loss. Polymeric flooring generally resists diluted acids and alkalis more effectively, although prolonged exposure to concentrated oxidizing acids, ketones, aromatic solvents, or chlorinated hydrocarbons may induce polymer swelling, oxidation, plasticization, or molecular chain scission [20,51,55].

### 4.5. Chemical Degradation

Chemical degradation frequently interacts with mechanical wear and moisture, accelerating overall deterioration. Consequently, compatibility between flooring chemistry and the expected industrial environment remains one of the most important design considerations. The combined effects of mechanical loading, substrate conditions, moisture, thermal cycling, chemical exposure, and UV radiation govern the long-term durability of flooring systems. The principal failure mechanisms, their primary causes, most affected flooring systems, associated engineering consequences, and typical mitigation strategies are summarized in Table 8.

## 5. Design and Selection Criteria for Advanced Flooring Systems

Selecting an appropriate flooring system requires an integrated engineering assessment in which material performance is matched to the specific operational, environmental, and economic demands of the facility. Modern industrial and commercial facilities impose diverse operational requirements, including high mechanical loading, aggressive chemical exposure, stringent hygienic standards, rapid installation schedules, and sustainability targets. Consequently, flooring systems should be selected through a comprehensive evaluation of service conditions, environmental exposure, maintenance strategy, expected service life, and economic constraints. In particular, life-cycle cost assessment can provide a more rational basis for material selection by accounting for initial installation, maintenance, repair, and replacement costs over the expected service period rather than considering initial material cost alone [97].

A successful flooring design requires balancing structural performance with operational functionality. While compressive strength and abrasion resistance are essential for logistics centers and manufacturing plants, thermal shock resistance becomes the governing criterion in food-processing industries, whereas chemical resistance and hygienic performance dominate pharmaceutical and healthcare facilities. Therefore, flooring specification should be regarded as a performance-based engineering process rather than a material-based selection. Several interrelated parameters should be evaluated during the design stage, including anticipated mechanical loading, traffic intensity, impact frequency, substrate condition, exposure to chemicals, cleaning procedures, temperature variations, moisture conditions, installation downtime, maintenance accessibility, and life-cycle cost. Failure to consider any of these parameters may significantly reduce flooring durability despite the use of high-performance materials [18,30].

Different industrial sectors impose distinct operational demands on flooring systems, requiring tailored material selection. Heavy manufacturing facilities typically prioritize abrasion resistance, compressive strength, and impact durability because floors are continuously subjected to forklifts, heavy machinery, and concentrated loads. In contrast, pharmaceutical plants and clean rooms emphasize seamless construction, cleanability, chemical resistance, and compliance with hygienic regulations. Food and beverage industries represent one of the most demanding environments because flooring must simultaneously resist thermal shock, organic acids, steam cleaning, moisture, and repeated sanitation procedures. Under such conditions, PU–cement flooring has become the preferred solution owing to its balanced combination of flexibility, chemical durability, and thermal stability [23,63].

In commercial, educational, healthcare, and retail environments, the need to balance aesthetics and occupant comfort with durability, safety, hygiene, and ease of maintenance typically governs flooring selection. In these environments, self-leveling epoxy systems, decorative PU flooring, or polyaspartic coatings may be selected depending on expected traffic and exposure conditions. Studies of healthcare flooring demonstrate that material selection should consider multiple performance criteria simultaneously, including indoor air quality, safety, infection control, cleanability, durability, and maintenance requirements [98]. Outdoor applications, including parking structures, pedestrian bridges, balconies, and transportation infrastructure, require excellent resistance to UV radiation and weathering. Polyaspartic and PMMA flooring systems are particularly advantageous because they combine rapid curing with high colour stability and environmental durability. Flooring selection should therefore be based on the specific service conditions of each application, including mechanical loading, chemical exposure, thermal conditions, hygiene requirements, environmental exposure, and operational constraints. Representative flooring technologies for major industrial and commercial applications, together with their key performance requirements, are summarized in Table 9.

Initial installation cost alone should not determine flooring selection. Although conventional concrete and thin epoxy coatings generally present lower initial costs, frequent maintenance, operational interruptions, and premature replacement may significantly increase the overall life-cycle cost. High-performance systems such as PU–cement, polyaspartic, and PMMA flooring typically require greater initial investment but often provide lower maintenance requirements, longer service life, and reduced operational downtime. Consequently, life-cycle cost analysis has become an increasingly important decision-making tool for flooring selection. A comprehensive economic assessment should consider not only material and installation costs but also maintenance frequency, repair expenses, operational interruptions, expected service life, energy consumption during installation, and end-of-life management [10,99,100]. Because flooring performance depends on multiple interacting criteria, material selection increasingly relies on multi-criteria decision-making approaches. Rather than optimizing a single property, engineers should simultaneously evaluate mechanical performance, durability, constructability, sustainability, and economic feasibility according to project-specific priorities.

## 6. Sustainability and Future Trends in Flooring Technologies

The construction sector represents a major source of global resource consumption, energy demand, and greenhouse-gas emissions, creating strong incentives for the development of more sustainable construction materials and technologies. Consequently, improving the sustainability of construction materials has become a major research priority, with flooring systems receiving increasing attention due to their extensive use in industrial and commercial buildings and their contribution to the overall environmental footprint of construction projects. For flooring systems specifically, environmental impacts can vary substantially with material composition, manufacturing requirements, installation, maintenance intensity, replacement frequency, and end-of-life strategy, highlighting the importance of evaluating the complete life cycle rather than the production stage alone.

Flooring materials were traditionally selected predominantly according to mechanical performance, durability, functional requirements, and initial installation cost. However, modern engineering practice increasingly incorporates environmental criteria such as embodied carbon, resource efficiency, indoor air quality, recyclability, maintenance requirements, and end-of-life management. As a result, the concept of sustainable flooring has evolved from simply extending service life to minimizing environmental impacts throughout the entire life cycle of the flooring system. Importantly, maintenance and replacement frequency can substantially influence overall environmental performance, meaning that a durable flooring system with a higher initial environmental burden may perform more favorably over an extended service life [101].

Environmental assessment requires separate treatment of embodied carbon, embodied energy, VOC emissions, resource use, service life, maintenance, replacement, and end-of-life impacts. These indicators are not interchangeable and may lead to different conclusions. Cross-system comparison is meaningful only when studies use compatible functional units, system boundaries, reference service lives, maintenance and replacement scenarios, and end-of-life assumptions [7,11,40].

Polymer-based flooring systems, including epoxy, PU, PMMA, and polyaspartic technologies, are primarily derived from petrochemical feedstocks and therefore possess relatively high embodied energy. However, their extended durability, low maintenance requirements, seamless construction, and superior protection of concrete substrates often reduce overall life-cycle impacts by extending service life and minimizing repair frequency [9]. Hybrid systems, particularly PU–cement flooring, frequently provide one of the most favourable balances between environmental performance and operational durability because their exceptional resistance to mechanical and chemical deterioration significantly extends service life under severe industrial conditions [23,28,36].

The transition toward a circular economy represents one of the most important future directions in flooring technology. Conventional flooring materials are often removed and disposed of at the end of service life, generating substantial quantities of construction and demolition waste. Consequently, increasing research efforts are focused on improving material recyclability, extending service life, and reducing dependence on virgin raw materials. Recent developments include the incorporation of recycled mineral aggregates, recycled glass, reclaimed rubber particles, recycled polymer fillers, and industrial by-products into both cementitious and polymer flooring systems. Simultaneously, bio-based polymers derived from renewable feedstocks such as vegetable oils, lignin, soybean oil, castor oil, and cardanol are increasingly investigated as sustainable alternatives to petroleum-derived resins [66,76].

**Figure 8:**
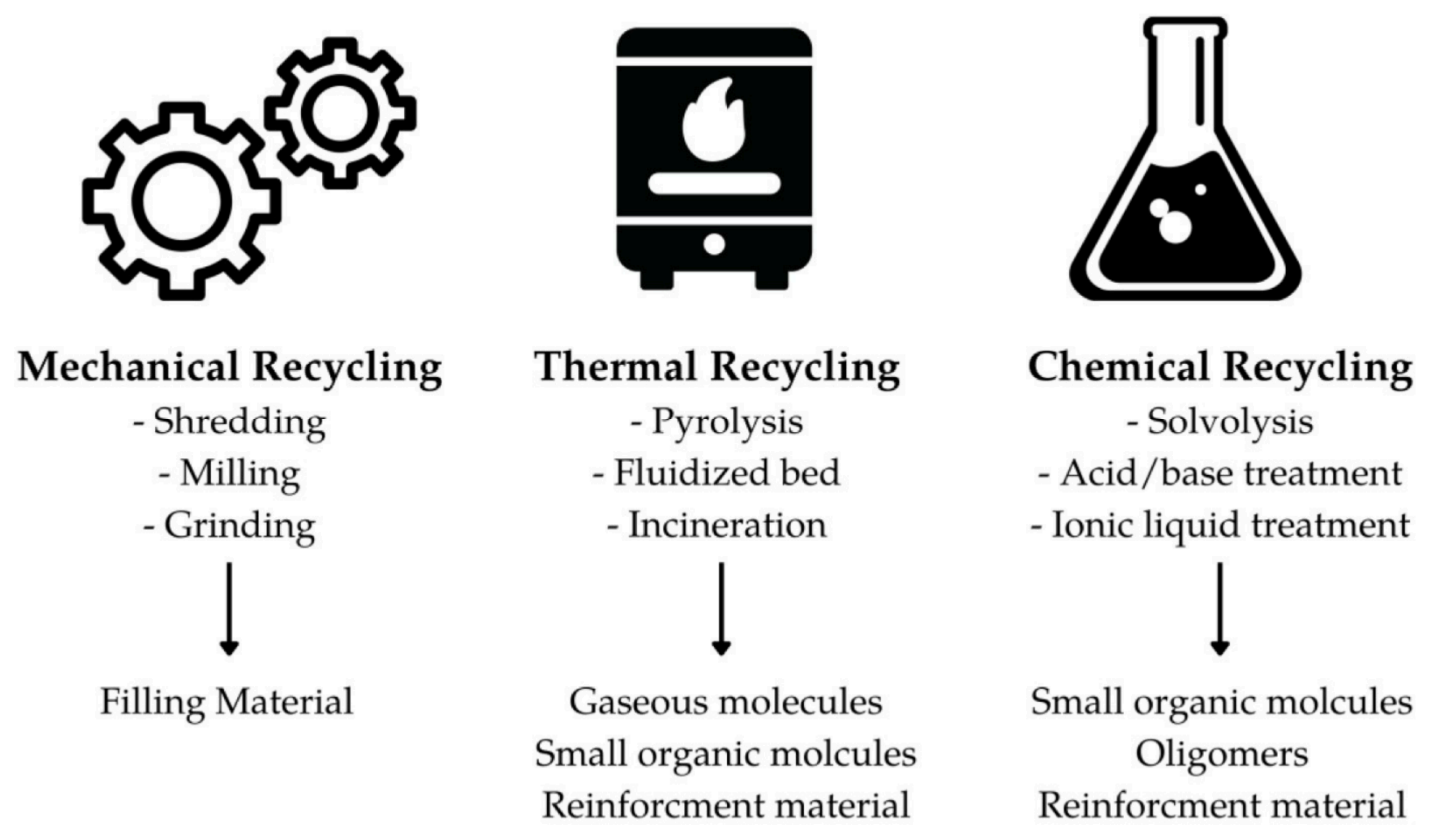
Conceptual circularity and recycling routes for epoxy-based thermosets: reproduced from [102], MDPI, 2023. Practical application to highly filled, concrete-bonded flooring remains constrained by separation, contamination, recovery, and processing requirements.

Recycling thermoset flooring is constrained by irreversible cross-linking and, in installed floors, by high mineral-filler content and intimate bonding to concrete. Separation, contamination, collection, transport, recovery yield, and the quality of recovered material can limit practical recycling. Vitrimer chemistry and chemical-recycling routes are promising research directions, but Figure 8 should not be interpreted as evidence that such routes are currently practical for highly filled, concrete-bonded floors [102].

Indoor environmental quality has become an increasingly important consideration in flooring specification, particularly in healthcare facilities, schools, offices, commercial buildings, and residential applications. Volatile organic compound emissions generated during installation and early curing may affect indoor air quality and occupational health [30]. Significant progress has been achieved through the development of solvent-free epoxy systems, low-VOC PU formulations, waterborne coatings, and improved curing technologies that substantially reduce emissions while maintaining high mechanical performance. Compliance with international environmental certification schemes has therefore become an important criterion in flooring selection for sustainable buildings [103,104].

**Figure 9:**
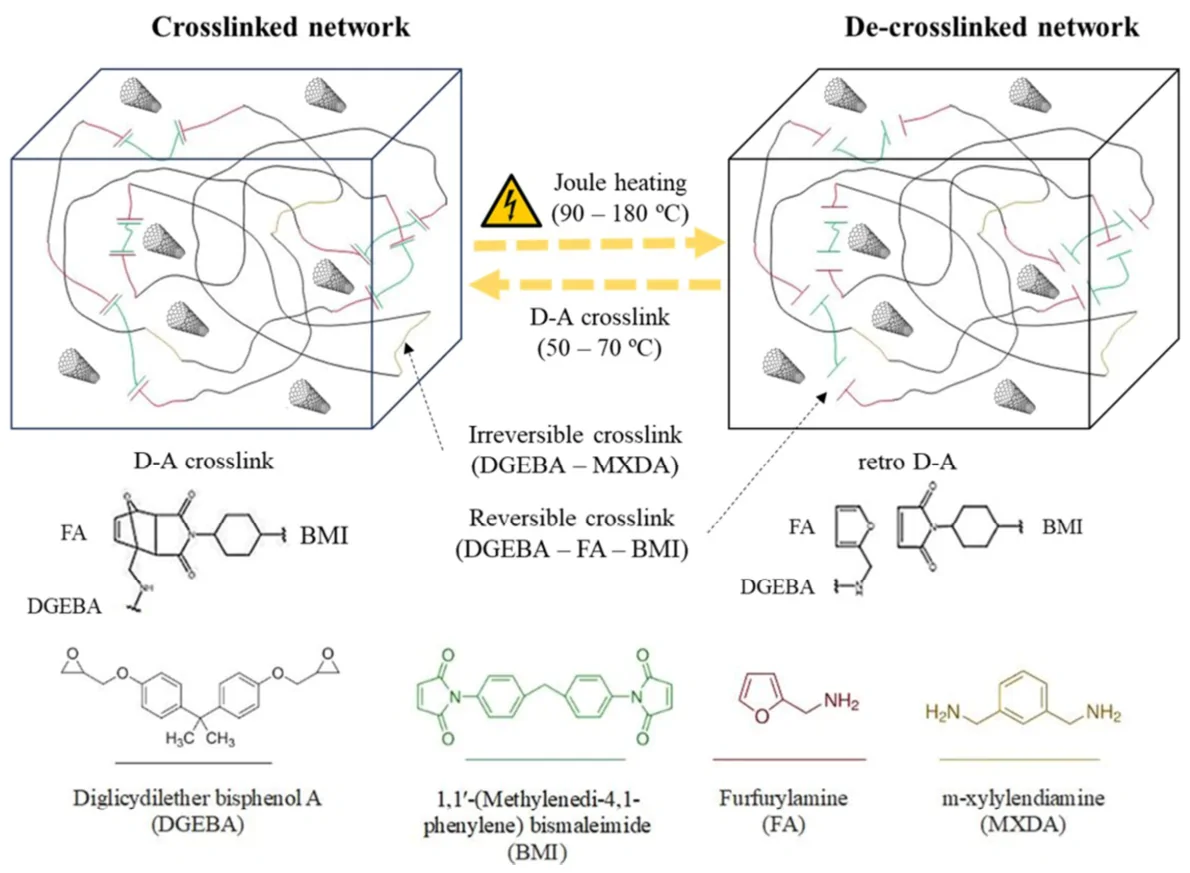
CNT/epoxy nanostructure and electrical functionality reported in broader polymer-composite research: reproduced from [105], MDPI, 2023. Flooring-specific performance is not established by this figure.

Nanomodified, self-healing, sensor-integrated, AI-assisted, and digital-twin approaches span different technology-readiness levels. The incorporation of carbon nanotubes (CNTs) into epoxy matrices represents a promising approach for introducing electrical conductivity and sensing functionality into polymer composites, as illustrated in Figure 9 [105]. However, the translation of these properties into industrial flooring applications requires further experimental validation under representative service conditions. Some sensing concepts have been demonstrated in smart-building or structural-monitoring contexts, whereas many self-healing and nanocomposite studies remain material- or laboratory-scale and are not validated as installed industrial floors. Translation requires evidence under realistic thicknesses, substrates, traffic, abrasion, chemicals, thermal and moisture exposure, installation constraints, repairability, data reliability, and long-term maintenance.

Because the reviewed evidence does not provide harmonized life-cycle inventories, functional units, thresholds, or system boundaries, categorical ratings such as High, Moderate, Very High, and sustainability potential are not used as comparative scores. Table 10 should instead be read only as a checklist of indicators requiring project-specific data: product-stage impacts, installation, VOC emissions, maintenance, service life, replacement, demolition, transport, and end-of-life treatment [72,105].

**Figure 10:**
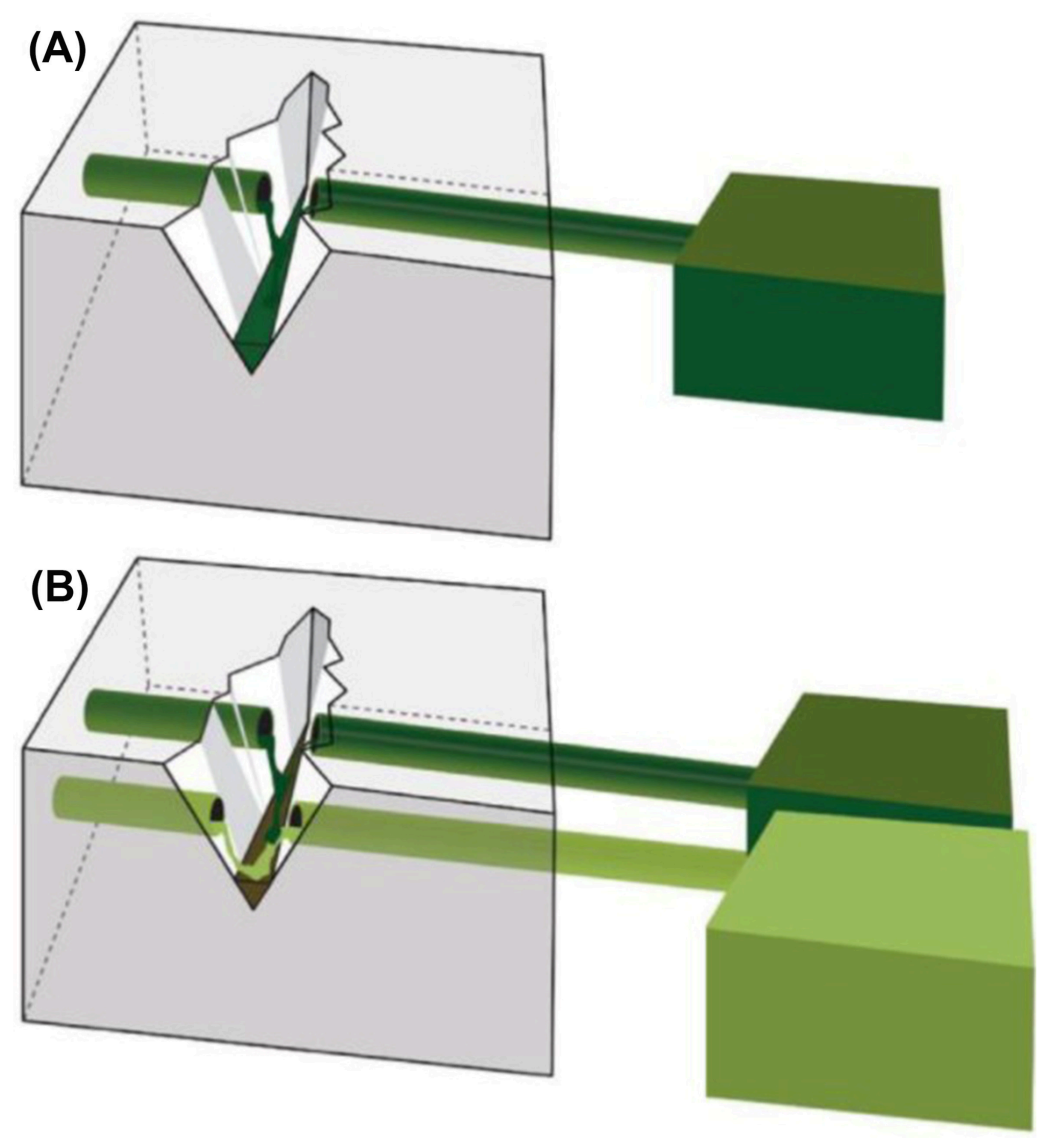
Schematic representation of vascular-based self-healing materials, illustrating the release and transport of healing agents from internal reservoirs to damaged regions through gravitational, capillary, and hydrostatic forces. Two vascular network configurations are presented: (**A**) a single-channel system and (**B**) a multi-channel system: reproduced from [101], MDPI, 2023. The mechanism remains to be validated under representative industrial-flooring substrates, loads, wear, and environmental exposure.

Self-healing polymers have emerged as a promising class of smart materials capable of autonomously repairing microdamage and thereby potentially extending service life while reducing maintenance requirements. Although most current developments remain at laboratory scale, microcapsule-based systems and dynamic covalent polymer networks have demonstrated promising results for future industrial flooring applications. In microcapsule-based systems, crack propagation ruptures embedded capsules and releases a healing agent into the damaged region, enabling restoration of structural or protective functionality without conventional external repair (Figure 10) [101,102,103].

Smart flooring systems integrating embedded sensors for temperature, strain, humidity, vibration, and traffic monitoring represent another rapidly developing research field. Combined with wireless communication and IoT technologies, these systems may enable continuous structural health monitoring and predictive maintenance throughout the service life of the flooring. Artificial intelligence and machine learning are also expected to transform flooring engineering by accelerating material formulation, predicting long-term durability, optimizing maintenance scheduling, and supporting performance-based material selection through data-driven decision-making [12,13]. The sustainability of flooring systems depends not only on their initial embodied carbon but also on service life, maintenance requirements, recyclability, and VOC emissions. A qualitative comparison of the principal sustainability indicators for cementitious and resin-based flooring technologies is presented in Table 10.

Despite substantial advances in materials design and manufacturing, important technical and methodological barriers still hinder the broader implementation of sustainable flooring technologies. Most published studies evaluate individual material properties under controlled laboratory conditions, whereas comprehensive investigations combining environmental exposure, mechanical loading, and long-term durability remain relatively scarce. In addition, standardized methodologies for comparing the life-cycle performance of different flooring technologies are still limited, making direct comparison among competing systems difficult. Indeed, life-cycle assessments of flooring materials have demonstrated that the environmental ranking of alternative systems can depend strongly on the selected assessment methodology, system boundaries, and life-cycle assumptions, emphasizing the need for harmonized evaluation frameworks [104].

Future advances in sustainable flooring are expected to arise from the convergence of low-impact material design, circular-economy strategies, smart sensing, and data-driven asset management. Research should focus on the development of low-carbon cementitious binders and bio-based polymer resins, together with recyclable thermosetting polymers and circular material design. Greater emphasis is also required on standardized life-cycle assessment methodologies and the long-term field validation of self-healing and smart flooring technologies. The integration of digital twins, IoT sensors, and AI could further support predictive maintenance and service-life optimization. By continuously integrating sensor-derived condition data with virtual models and predictive algorithms, digital-twin frameworks can facilitate condition-based maintenance, early detection of deterioration, and more informed maintenance decisions throughout the service life of building components [105]. In parallel, multi-scale modelling and performance-based design approaches should link material chemistry and microstructure with durability and structural performance while simultaneously considering environmental, economic, and engineering criteria.

## 7. Conclusions

The evidence indicates that flooring selection is conditional on service environment and system configuration. Cementitious systems primarily provide structural capacity; epoxy systems can provide strong bond and chemical protection under suitable conditions; PU and PU–cement systems may be advantageous where deformation capacity and defined thermal cycling are important; and PMMA or polyaspartic polyurea systems can reduce return-to-service time in appropriate formulations. Direct ranking remains limited by differences in formulations, specimens, thicknesses, standards, exposure, and reporting. Unresolved issues include coupled degradation, long-term interface durability, comparable life-cycle data, and field validation.

Future work should prioritize flooring-specific standardized tests, multi-factor ageing, transparent reporting of formulation and layer configuration, field validation, and life-cycle assessment with defined functional units, boundaries, maintenance, replacement, and end-of-life scenarios. Smart sensing, self-healing materials, nanomodification, AI, and digital twins remain promising, but require validation under realistic flooring loads, exposures, installation constraints, and maintenance regimes.

## Figures and Tables

**Table 1:** The main properties of flooring systems are discussed throughout this review.

Flooring Category	Main Binder/Material	Typical Thickness	Key Advantages	Main Limitations	Typical Applications
Cementitious	Portland cement	Structural slabs: project-specific; screeds/toppings: system-specific	High compressive strength, economical	Cracking, dusting, chemical sensitivity	Warehouses, factories
Epoxy	Epoxy resin	0.5–6 mm	Excellent adhesion, chemical resistance	Brittle behavior, UV sensitivity	Pharmaceutical, electronics
PU	PU resin	2–9 mm	Flexibility, thermal shock resistance	Higher cost	Food processing, cold storage
PMMA	Acrylic resin	2–6 mm	Rapid curing, low downtime	Odor during installation	Retail, logistics
Polyaspartic	Polyaspartic polyurea	0.5–4 mm	UV stability, fast curing	Higher material cost	Parking structures, commercial buildings
Hybrid PU–Cement	Cement + PU	4–12 mm	Excellent durability, thermal resistance	Specialized installation	Heavy industry
Vinyl/PVC	Thermoplastic polymer	2–5 mm	Comfort, aesthetics	Lower mechanical resistance	Offices, healthcare
Rubber	Elastomer	3–10 mm	Slip resistance, impact absorption	Chemical limitations	Gyms, schools
Ceramic/Stone	Ceramic/Natural stone	8–25 mm	Excellent wear resistance	Brittle joints	Commercial buildings
Terrazzo	Cement or resin	15–30 mm	Long service life, decorative finish	Skilled installation	Airports, public infrastructure

**Table 2:** Principal constituents of cementitious flooring systems and their roles in governing mechanical, rheological, and durability-related performance.

Constituent	Primary Function	Effect on Flooring Performance
Portland cement	Hydraulic binder	Strength development
Fine aggregates	Packing density	Surface finish
Coarse aggregates	Load transfer	Abrasion resistance
Water	Hydration medium	Workability and porosity
Superplasticizers	Flowability	Lower w/c ratio
Silica fume	Pozzolanic reaction	Higher density and durability
Fly ash	Secondary hydration	Improved workability and lower heat
Ground granulated blast-furnace slag	Latent hydraulic activity	Sulfate resistance and lower CO_2_
Fibers	Crack bridging	Toughness and fatigue resistance
Surface hardeners	Wear-resistant layer	Abrasion resistance

**Table 3:** Performance comparison between cementitious and resin flooring materials.

Property	Cementitious Flooring	Resin Flooring
Structural contribution	Qualitative; project-specific verification required	Low
Thickness	Not directly comparable; distinguish slabs, screeds, and toppings	0.5–12 mm
Chemical resistance	Moderate	Qualitative; verify for the specified formulation and exposure
Abrasion resistance	Qualitative; project-specific verification required	Qualitative; verify using a stated criterion and test method
Hygienic performance	Moderate	Qualitative; verify for the specified formulation and exposure
Seamless finish	No	Yes
Repairability	Moderate	Qualitative; project-specific verification required
UV stability	Qualitative; project-specific verification required	Resin-dependent
Installation speed	Moderate	High (PMMA, Polyaspartic)
Typical service life	20–40 years	15–30+ years

**Table 4:** Typical mechanical, adhesive, durability, and service-performance characteristics of epoxy flooring systems.

Property	Typical Performance
Compressive strength	Configuration- and test-dependent; distinguish resin, filled mortar, and installed system
Tensile strength	Configuration- and test-dependent; distinguish resin, filled mortar, and installed system
Elastic modulus	Configuration- and test-dependent
Adhesion to concrete	Often exceeds concrete tensile strength
Abrasion resistance	Qualitative; verify for the specified formulation and exposure
Chemical resistance	Qualitative; verify for the specified formulation and exposure
UV stability	Moderate to poor (without topcoat)
Thermal shock resistance	Moderate
Typical service life	15–25+ years

**Table 5:** Principal failure mechanisms of epoxy flooring systems, their underlying causes, microstructural effects, engineering consequences, and corresponding mitigation strategies.

Failure Mechanism	Primary Cause	Microstructural Effect	Engineering Consequence	Mitigation Strategy
Adhesive failure	Poor surface preparation	Loss of interfacial bonding	Delamination	Shot blasting, primers
Cohesive failure	Weak concrete substrate	Crack propagation within concrete	Local failure	Higher substrate strength
Osmotic blistering	Residual moisture	Vapor pressure buildup	Blister formation	Moisture barriers
UV degradation	Photo-oxidation	Chain scission, oxidation	Yellowing, embrittlement	UV-resistant topcoats
Thermal ageing	Differential thermal expansion	Residual stresses	Cracking	Flexible overlays
Abrasion	Rolling traffic	Progressive material removal	Wear	Quartz or ceramic fillers
Chemical degradation	Acids, solvents	Polymer degradation	Softening, loss of strength	Chemical-resistant formulations

**Table 6:** Typical performance characteristics and application fields of PMMA flooring systems.

Property	PMMA Flooring
Curing time	Condition-dependent; identify access and serviceability criterion
UV resistance	Qualitative; verify for the specified formulation and exposure
Abrasion resistance	Qualitative; verify for the specified formulation and exposure
Chemical resistance	Qualitative; verify for the specified formulation and exposure
Low-temperature application	Qualitative; verify for the specified formulation and exposure
Flexibility	Moderate
Typical thickness	2–6 mm
Typical applications	Cold storage, parking decks, food industry, logistics

**Table 7:** Typical performance characteristics and application fields of polyaspartic flooring systems.

Property	Typical Performance
Curing time	2–6 h
UV resistance	Qualitative; verify for the specified formulation and exposure
Abrasion resistance	Qualitative; verify for the specified formulation and exposure
Chemical resistance	Qualitative; project-specific verification required
Flexibility	Moderate–High
Typical thickness	0.5–4 mm
Typical applications	Parking decks, airports, bridges, commercial buildings

**Table 8:** Principal failure mechanisms of industrial and commercial flooring systems, their primary causes, most affected flooring technologies, engineering consequences, and typical mitigation strategies.

Failure Mechanism	Primary Cause	Most Affected Systems	Engineering Consequence	Typical Mitigation
Abrasion	Rolling traffic	All systems	Surface wear	Hard aggregates, ceramic fillers
Interfacial debonding	Poor surface preparation	Resin flooring	Delamination	Shot blasting, primers
Moisture damage	Water ingress	Cementitious, epoxy	Blistering, adhesion loss	Moisture barriers
Thermal cracking	Thermal cycling	Concrete, epoxy	Cracking	Flexible systems, movement joints
Chemical attack	Acids, solvents	Cementitious	Material degradation	Chemical-resistant flooring
UV ageing	Solar radiation	Aromatic epoxy	Yellowing, chalking	UV-stable topcoats
Fatigue	Cyclic loading	All systems	Progressive cracking	Toughened formulations

**Table 9:** Recommended flooring systems for representative industrial and commercial applications according to key performance requirements.

Application	Recommended Flooring	Key Performance Requirements
Warehouses and logistics centres	Epoxy mortar, dry-shake concrete	Abrasion resistance, compressive strength
Food processing	PU–cement	Thermal shock, hygiene, chemical resistance
Pharmaceutical industry	Self-leveling epoxy	Seamless surface, cleanability, chemical resistance
Commercial kitchens	PU–cement	Steam cleaning, impact resistance
Hospitals	Self-leveling epoxy or PU	Hygiene, low VOC, easy maintenance
Parking garages	Polyaspartic, PMMA	UV resistance, rapid curing, weatherability
Electronics manufacturing	ESD epoxy	Electrostatic dissipation
Airports and transport terminals	Polyaspartic, PMMA	Abrasion resistance, rapid return to service

**Table 10:** Comparative sustainability indicators of cementitious and resin-based flooring systems.

Criterion	Cementitious	Epoxy	PU	PMMA	Polyaspartic	PU–Cement
Embodied carbon	Qualitative; project-specific verification required	Moderate	Moderate	Moderate	Moderate	Moderate
Service life	Qualitative; project-specific verification required	Qualitative; project-specific verification required	Qualitative; project-specific verification required	Qualitative; project-specific verification required	Qualitative; project-specific verification required	Qualitative; verify using a stated criterion and test method
Maintenance demand	Moderate	Low	Low	Low	Low	Very Low
Recyclability	Moderate	Limited	Limited	Limited	Limited	Moderate
VOC emissions	Low	Low–Moderate	Low	Moderate	Low	Low
Sustainability potential	Moderate	Qualitative; project-specific verification required	Qualitative; project-specific verification required	Qualitative; project-specific verification required	Qualitative; project-specific verification required	Qualitative; verify using a stated criterion and test method

## Data Availability

No new data were created or analyzed in this study. Data sharing is not applicable to this article.

## Abbreviations

The following abbreviations are used in this manuscript:

AI	Artificial Intelligence
CMF	Cellulose Microfibers
CNCs	Cellulose Nanocrystals
CNFs	Cellulose Nanofibers
CNTs	Carbon Nanotubes
ESD	Electrostatic Discharge
IoT	Internet οf Things
LCA	Life Cycle Assessment
MCC	Microcrystalline Cellulose
mCMFs	Modified Cellulose Microfibers
PMMA	Polymethyl Methacrylate
PU	Polyurethane
T_g_	Glass-Transition Temperature
UHPC	Ultra-High-Performance Cementitious Flooring
UV	Ultraviolet
VOCs	Volatile Organic Compounds
